# *In vivo* Mouse Intervertebral Disc Degeneration Models and Their Utility as Translational Models of Clinical Discogenic Back Pain: A Comparative Review

**DOI:** 10.3389/fpain.2022.894651

**Published:** 2022-06-22

**Authors:** Shirley N. Tang, Benjamin A. Walter, Mary K. Heimann, Connor C. Gantt, Safdar N. Khan, Olga N. Kokiko-Cochran, Candice C. Askwith, Devina Purmessur

**Affiliations:** ^1^Department of Biomedical Engineering, The Ohio State University, Columbus, OH, United States; ^2^Department of Orthopaedics, Wexner Medical Center, The Ohio State University, Columbus, OH, United States; ^3^Department of Neuroscience, The Ohio State University, Columbus, OH, United States; ^4^Institute for Behavioral Medicine Research, Neurological Institute, The Ohio State University, Columbus, OH, United States

**Keywords:** mouse model, intervertebral disc (IVD), discogenic back pain, pain behavior assessment, cellular and molecular, structure and function analysis

## Abstract

Low back pain is a leading cause of disability worldwide and studies have demonstrated intervertebral disc (IVD) degeneration as a major risk factor. While many *in vitro* models have been developed and used to study IVD pathophysiology and therapeutic strategies, the etiology of IVD degeneration is a complex multifactorial process involving crosstalk of nearby tissues and systemic effects. Thus, the use of appropriate *in vivo* models is necessary to fully understand the associated molecular, structural, and functional changes and how they relate to pain. Mouse models have been widely adopted due to accessibility and ease of genetic manipulation compared to other animal models. Despite their small size, mice lumbar discs demonstrate significant similarities to the human IVD in terms of geometry, structure, and mechanical properties. While several different mouse models of IVD degeneration exist, greater standardization of the methods for inducing degeneration and the development of a consistent set of output measurements could allow mouse models to become a stronger tool for clinical translation. This article reviews current mouse models of IVD degeneration in the context of clinical translation and highlights a critical set of output measurements for studying disease pathology or screening regenerative therapies with an emphasis on pain phenotyping. First, we summarized and categorized these models into genetic, age-related, and mechanically induced. Then, the outcome parameters assessed in these models are compared including, molecular, cellular, functional/structural, and pain assessments for both evoked and spontaneous pain. These comparisons highlight a set of potential key parameters that can be used to validate the model and inform its utility to screen potential therapies for IVD degeneration and their translation to the human condition. As treatment of symptomatic pain is important, this review provides an emphasis on critical pain-like behavior assessments in mice and explores current behavioral assessments relevant to discogenic back pain. Overall, the specific research question was determined to be essential to identify the relevant model with histological staining, imaging, extracellular matrix composition, mechanics, and pain as critical parameters for assessing degeneration and regenerative strategies.

## Introduction

Low back pain (LBP) is the leading cause of disability worldwide and its prevalence continues to increase with enormous socioeconomic burdens exceeding $100 billion annually in the United States alone (1–3). Current clinical interventions include analgesics (i.e., non-narcotic pain medications, non-steroidal anti-inflammatory drugs (NSAIDs), and opioids), physical therapy, epidural injections, and surgical interventions (4–6). While most of these treatments provide symptomatic pain relief, they do not target the underlying pathology, leading to recurrent pain and surgical interventions (6). This ultimately leads to the increased use of pain medications and contributes significantly to the escalating opioid crisis (7).

Epidemiological studies suggest intervertebral disc (IVD) degeneration is a major cause of LBP, attributing to 40% of all LBP cases (8, 9). It is important to note here that the nomenclature “IVD degeneration” refers to the progressive disease characterized by cellular and matrix changes which can often, but not always, result in disc herniation with increasing severity. Disc herniation can also occur due to injury/trauma to the spine resulting in mechanical compression on nerve roots (10). The IVD is a fibrocartilaginous structure connecting vertebral bodies which plays critical roles in everyday motion, however, the etiology of IVD degeneration is a complex, multifactorial process contributing to discogenic back pain (DBP) (11). While several studies have explored regenerative therapies to treat disc degeneration, their clinical translation may be hampered by a lack of *in vivo* animal models that combine ***all*** the cellular, structural and functional aspects of IVD degeneration including symptomatic pain. Individual models recapitulate different aspects of IVD degeneration and different results are often gained when different models are used.

Numerous pre-clinical models have been used to assess therapies for IVD regeneration ranging from large (sheep, goats, pigs, cattle, canine) to small (rabbits, rats, mice) animals. *In vivo* models in particular are advantageous over *in vitro* models as they incorporate complex systemic interactions which are relevant for assessing pain/cognitive behaviors that are otherwise difficult to recapitulate *in vitro* (12). Many of these *in vivo* models have been discussed in detail in numerous review articles which cover advantages and disadvantages along with major findings (12–17). The goal and novel aspect of this review are to summarize relevant similarities and differences across mouse models and human IVD degeneration with an emphasis on evaluating the clinical translation of downstream parameters and the metrics of pain. Rodent models such as mice are widely used and are advantageous in their accessibility, affordability, tunability, and abundance of molecular tools/genomic databases compared to other large animal models (12). While many studies have used mouse models of IVD degeneration to test therapies for DBP, to enhance clinical translation, standardization of key variables ranging from the method of model induction to the downstream parameters measured may be beneficial. In particular, there are diverse array of pain-like behavior tests available and challenges in the interpretation of these behaviors across numerous studies also highlights the need to ground the choice of relevant assays in translation to the human condition. In addition, many studies differ in the age and sex of animals used which critically influence IVD degeneration and the pathogenesis of pain both in mice and humans (18–20). The lack of standardization in which parameters are assessed across models may hamper the clinical translatability of using mouse as a translational model for regenerative therapies. Often the differing array of parameters used across models relate to the specific tools/skill set investigators have readily available to them, pointing toward a need to consider multi-disciplinary teams as critical to components of our *in vivo* animal model studies.

Thus, this review aims to (**1)** highlight current/existing mouse models of IVD degeneration for translational research as it relates to human LBP along with their advantages and limitations, (**2)** compare/contrast degeneration induction methods and outcomes between these different mice models, and (**3)** assess the clinical relevance of these outcomes to identify a subset of critical/core parameters essential for validating both the model and its utility to screen regenerative therapies to treat LBP. The following sections will review current mice models, the different levels of assessments used across studies, how these assessed parameters compare with the clinical human condition and conclude with critical assessment parameters.

## Mice Models of Intervertebral Disc Degeneration

### The Human Intervertebral Disc vs. Mouse Intervertebral Disc

The human IVD is comprised of three main components: the inner nucleus pulposus (NP), the surrounding annulus fibrosus (AF), and the superior and inferior cartilage endplates (CEP) (21). The healthy NP is highly gelatinous and comprised of proteoglycans, namely aggrecan [i.e., glycosaminoglycan (GAG)], and collagen II which provides load distribution and compressive force absorption (22, 23). In contrast, the surrounding AF region is comprised of an aligned matrix of collagen I fibers and functions to contain the NP and anchor the disc to adjacent vertebrae to resist torsional and tensional loads (24, 25). While under characterized, the CEP plays a critical role in the diffusion of nutrients to the largely avascular IVD from the vertebral bodies as well as the removal of metabolic waste (26). However, with advancing degeneration, the IVD demonstrates decreases in cellularity and proteoglycan synthesis along with increased catabolism, inflammation, immune cell infiltration, changes in IVD structure, altered mechanics, and neurovascular invasion, all of which are critical factors to assess in IVD degeneration models (27–30).

**Figure 1:**
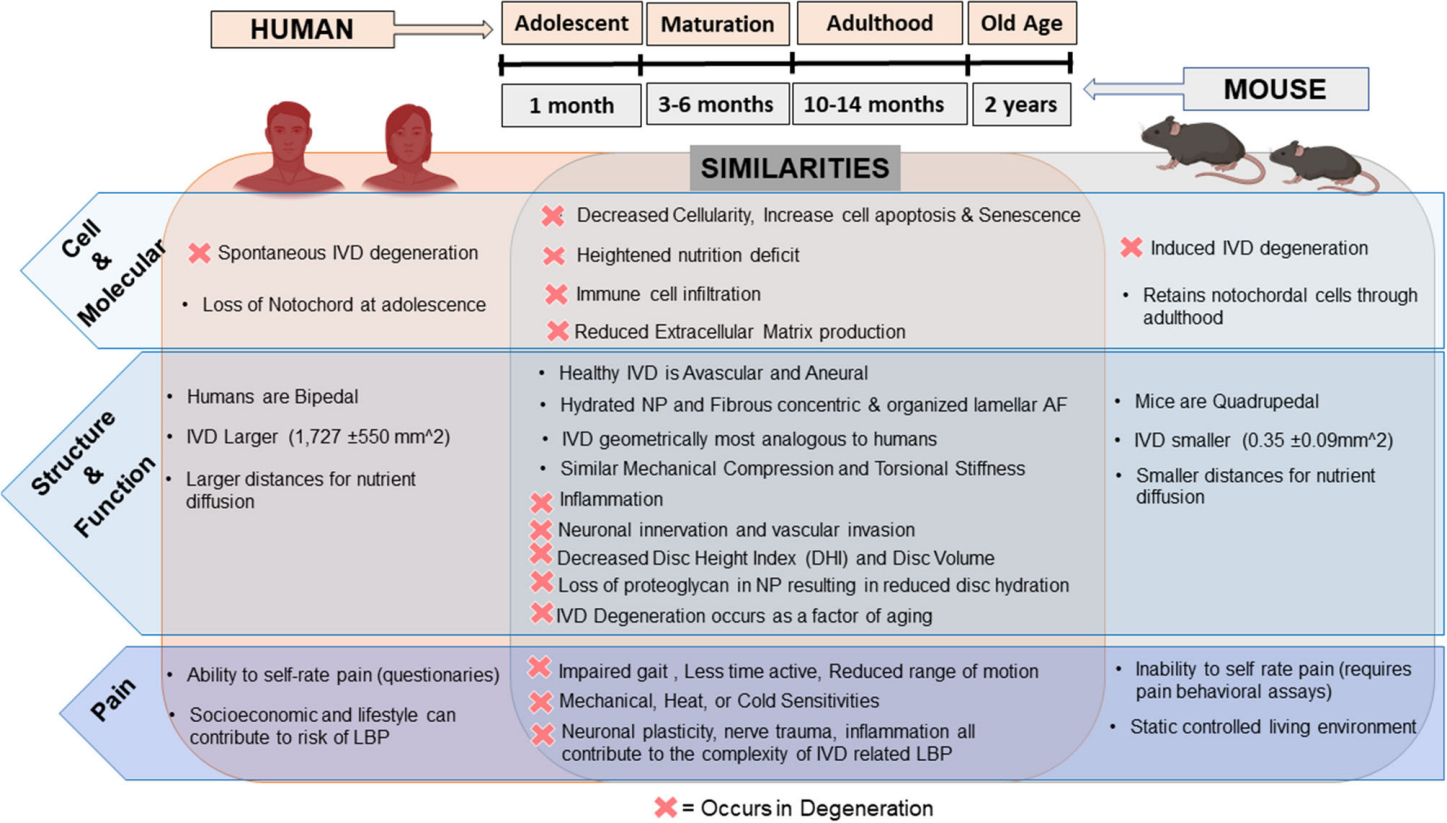
Comparison of the human vs. mice intervertebral disc and changes in degeneration: humans are represented on the left along with mice on the right and their respective similarities in the healthy and degenerate/diseased IVD (D = occurrence in degeneration) overlapping in the middle. Top middle depicts relative timeline of mice age compared to humans. Depiction of human and mice in this figure generated using BioRender.com.

In comparison to the human IVD, mice, despite their small stature, have many commonalities with humans as summarized in Figure 1. On the cellular level, humans and mice IVDs both exhibit decreased cellularity with increased apoptosis, senescence, and immune infiltration during aging or induced IVD degeneration (13, 31). At a molecular level, nutrient deficiency, and reduced extracellular matrix (ECM) production have been found in mouse models of IVD degeneration and degenerated human IVDs. Immune infiltration has been identified in painful degenerate human and mouse IVDs as evidenced by the presence and recruitment of mast cells and macrophages (32–37). Structurally, human and mouse IVDs are avascular and aneural in their healthy state with similar components (NP, AF, CEP) and ECM tissue structure. Interestingly, rodent lumbar IVDs are most geometrically analogous to humans compared to other animals based on percent deviation of normalized disc height, anterior-posterior width, and NP area (38). In IVD degeneration, changes at the structure/function level in both humans and mice include decreased disc height/volume, neurovascular invasion, limited nutrition, reduced hydration, AF disorganization, and a more fibrous NP (38–42). In terms of pain and pain-like behaviors, humans and mice can experience impaired gait, decreased activity time, and reduced range of motion due to IVD degeneration along with mechanical and thermal hypersensitivity and changes in neuronal plasticity (43–47).

In contrast, drawbacks of using mice as a translational model of LBP are mainly due to their small size, low forces and reduced diffusion distances across their IVD (0.35 ± 0.09 mm^2^), quadrupedal nature with altered mechanical forces, higher cellularity, and retention of notochordal cells throughout adulthood (13, 38). Ideally, research involving therapeutic studies should include quantification of pain, but the measurement of pain differs from human patients to mice as animals cannot communicate pain levels and are not affected by other risk factors of LBP such as lifestyle and career occupation (48). Instead, behavioral assessments for “pain-like” behaviors are used and inferred. Nonetheless, mouse models allow for pre-clinical validation of disease pathogenesis and evaluation of potential regenerative therapies and therefore remain critical *in vivo* research models. The existing mouse models can be categorized into aging and genetic, mechanically induced and puncture models.

### Age-Related Spontaneous Degeneration and Genetic Models

Age-related changes representative of degeneration are not as severe in mice, as they retain their notochordal cell population throughout adulthood, compared to animals without notochordal cell retention such as primates and chondrodystrophic dogs (49). While less severe, mice do develop age related changes at later stages of life. Specifically, wild-type C57BL/6J mice at 2 years old (old age) demonstrate features of IVD degeneration such as reduced disc height, bulging, and neurovascular invasion in both caudal and lumbar IVDs along with upregulation of inflammatory cytokines tumor necrosis factor-alpha (TNFα) and interleukin-1 beta (IL-1β) compared to younger mice (50). Alvarez et al. also showed degeneration at 2 years in wild-type mice associated with downregulated Forkhead box O (FOXO) expression (51). Taken together, aging in wild-type mice may mimic IVD degeneration that occurs with age in humans and has high clinical relevance. However, laboratory mice typically have a lifespan of 2 years, potentially limiting the utility of using aged mice for studying regenerative therapies as they may not survive the length of treatment.

As such, genetically modified mice have been used in place of wild-type mice for “accelerated aging” or for studying specific mechanisms involved in the pathogenesis of IVD degeneration. The bile duct ligation (BDL) strain of mice, a result of an autosomal mutation in the *ky* gene, develop kyphoscoliosis with structural changes in the vertebrae and cervical IVDs coupled with degeneration and herniation (52). Growth differentiation factor 5 (GDF5) deficient mice also demonstrate signs of IVD degeneration with changes in collagen and proteoglycan content (53). In addition, secreted protein acidic and rich in cysteine-null (SPARC-null) transgenic mice demonstrate age-related changes in IVD composition and structure such as decreased disc height index (DHI) and GAG with increasing severity. SPARC-null mice also exhibit similar human pain-like behaviors such as decreased tolerance to axial stretch, thermal sensitivity, and impaired locomotion (54). More mouse models that stimulate an “age-related” degenerative phenotype include Excision Repair 1 (ERCC1)-mutant mice that lack important DNA repair mechanisms, suggesting aberrant DNA repair may contribute to IVD degeneration (55). Most recently, SM/J mice have been shown to exhibit spontaneous IVD degeneration due to poor cartilage healing (56). As degradation of the ECM plays a key role in the pathogenesis of IVD degeneration, many ECM genetic knockout models have also been assessed (i.e., collagen II, collagen IX, aggrecan, and biglycan) and as expected demonstrate significant ECM loss/disorganization with impaired disc and vertebrae development (57–62). A review by Jin et al. highlights additional genetic knockouts with degenerative IVD phenotypes (12). While specific mutations have been identified in some populations and global deletions remain rare, genetic models demonstrate accelerated disease/aging which is of clinical relevance and value for animal studies given how long degeneration can take to progress in humans (63–65).

Age-related models are advantageous as they share many similarities with disease pathogenesis that occurs in degenerate human IVDs. However, it is important to note that IVD degeneration does not happen solely due to age, but results from multiple factors such as genetics, environment, and lifestyle; furthermore, IVD degeneration can also occur in the younger population (63, 65, 66). Limitations of using aged mice as a model for regenerative therapies include the long-term care of aging mice and the short lifespan of the mice. Genetic mouse models are excellent tools allowing the investigation of specific genes/proteins/pathways contributing to IVD degeneration. Limitations of these models may include the multifactorial disease process that occurs in humans which may not be gene-specific, however, these models still allow for screening of novel regenerative therapies for IVD degeneration.

### Mechanically Induced and Puncture Models

Mechanical loading is an important regulator of IVD development and homeostasis and altered biomechanics plays a significant role in IVD degeneration in humans as evidenced by the increased risk of IVD degeneration in manual laborers and changes due to physical exercise (9). As such, a wide array of mechanically induced models have been developed that alter or modify the mechanical environment experienced by mice IVDs to induce degeneration. Instability models such as surgical resection of posterior elements (i.e., facet joints and spinous processes), tail bending, static/dynamic compression, and axial loading models have demonstrated that altering the mechanical loading environment applied to the IVD can lead to progressive disc degeneration characterized by cell death, decreased disc height, decreased collagen II and aggrecan expression, and disorganization of AF structure (67–74). Other models which alter spinal loading *via* induced bipedal behaviors exhibit accelerated degenerative characteristics due to abnormal mechanical stress compared to quadrupedal mice (75, 76). A recent bipedal mice model was established by placing mice in water to promote standing as, mice are aqua-phobic, and have demonstrated decreased DHI with increasing degeneration grade (76). Reduced spinal loading due to microgravity, investigated through space flight, has also been shown to alter the viscoelastic behaviors of caudal mouse IVDs upon return to earth (77). A model of whole-body vibration has also induced structural changes in mice IVD indicated by structural NP and AF deficits (78). Collectively, these models are capable of inducing degeneration by manipulating the external loads applied to the IVD and these models demonstrate high clinical relevance given the significant changes and enhanced stresses and loads experienced by humans with degeneration and aging.

Another common method to induce IVD degeneration is *via* needle puncture, which induces a direct injury to the IVD and is thought to depressurize the NP, thereby changing the normal internal mechanisms of load support (79, 80). Needle puncture models have shown similar hallmarks of IVD degeneration as in humans including decreased DHI, reduced disc hydration and cellularity, matrix disorganization, and reduced torsional and compressive stiffness (79, 81–86). The severity of degeneration can be influenced by multiple factors such as needle gauge, surgical procedure, depth of puncture, and region of injury (i.e., caudal vs. lumbar) (40). There has been considerable work in multiple animal models to determine how the size of the puncture relative to IVD height influences degeneration (79, 87–90). Across models, there appears to be a threshold in which a smaller injury ratio (i.e., needle diameter to disc height ratio) induces less severe degeneration and above which severe degeneration is induced, however, the exact threshold varies based on the species and region of the puncture (87, 90). In mice, an injury ratio of 90% (26G needle) significantly altered IVD compressive and torsional mechanics while a 65% injury ratio (29G needle) had no significant differences (79). It is important to note that puncture models are useful due to the acute or rapid degeneration of the punctured discs. However, the puncture itself is generally not intentionally induced in humans in the present day.

Mechanical and puncture induced models are advantageous as they do not require significantly aged mice or genetic modification to induce accelerated degeneration. However, it is worth noting that the severity of degeneration relies heavily on the degree of mechanical loading or injury. To date, puncture models have primarily been induced in caudal level IVDs due to surgical accessibility. However, lumbar level puncture models may be more representative of LBP and give the capability to assess pain-like behaviors. In addition, injury induced degeneration often results in post-injury inflammatory processes which may be mechanistically different from spontaneous IVD degeneration and more representative of acute trauma (91). Thus, the type and degree of degeneration that the therapeutic strategy aims to target are critical for determining the relevant animal model utilized.

### Miscellaneous Models

Other models exist such as dietary-induced diabetic or Tobacco smoke inhalation models which demonstrate increased disc degeneration with increases in cell senescence and reduced proteoglycan synthesis compared to control mice not exposed to a high-fat diet or tobacco products (92, 93). Multiple studies have also shown that diabetes and obesity are significant risk factors for IVD degeneration which may serve as models if studying diabetic/obesity-related IVD degeneration (94–98).

### Methodology and Scope of Review

To focus the scope of this comparative review on translational mice models of LBP for studying regenerative therapies, articles were collected *via* PubMed and Scopus with multiple combinations of the search terms: “Intervertebral Disc,” “Low Back Pain,” “Mice,” “Mouse,” “Model,” “Discogenic back pain” and “degeneration.” Articles included in the review met the following criteria: characterization of a mouse model, direct/indirect induction of IVD degeneration (lumbar or caudal), and potential for use as an IVD degeneration model for regenerative medicine (i.e., no genetic deficits that greatly alter other tissues/organs). Exclusion criteria included: studies with a focus on specific pathologies/mechanisms or spinal diseases beyond IVD degeneration (i.e., Scoliosis, kyphosis, arthritis) or LBP unrelated to IVD degeneration (i.e. direct neuronal trauma without IVD degeneration). Respective articles assessing Molecular and Cellular changes (Table 1), Tissue & Structure/Function (Table 2), and Pain-like behavior (Table 3) were compared for their induction methods (aging, injury type, degeneration level (i.e., caudal vs lumbar), length of study, model demographics (age, sex, strain), along with study results. The following sections will review different assessments to identify key parameters for translation to the human condition.

**Table 1:** Comparison of mouse models and their molecular and cellular assessments.

									**Molecular and Cellular assessments**				
**Model type**	**Paper title (reference)**	**Model details**	**Strain**	**Sample size per assess-ment**	**Sex**	**Age**	**Levels affected and or assessed**	**Timeline**	**Phenotypic markers and inflammatory cytokines**	**Cell morphology**	**Cellularity**	**Cell specific ECM markers**	**Matrix catabolism**
**Mechanically Induced**	Compression-induced degeneration of the intervertebral disc: an *in vivo* mouse model and finite-element study (99)	Compression device with pins through adjacent vertebrae with stress less of 0.4, 0.8 or 1.3 MPa or SHAM for 1 week and allowed to recuperate for 1 month	Swiss Webster mice	*N* = 3–19	Male	12 weeks	C9-C10	1 week and 1 month loading	–	**•Histology** (H&E, Safranin O): Disaggregation of notochordal cells after 1 week	**•TUNEL:** low compression saw ↑cell death in NP region and inner AF and spread into outer AF and CEP with higher compression	**•*****in-situ*** **Hybridization:** ↓Col1a expressing cells after 1 week. No change in ACAN	–
	Repeated exposure to high-frequency low-amplitude vibration induces degeneration of murine intervertebral discs and knee joints (78)	Vertical sinusoidal vibration (for mimicking humans whole-body vibration)–> 30 min per day, 5 days/week, 45 Hz with peak acceleration at 0.3 g	CD-1	*N* = 4–6	Male	10 weeks	Whole Body Vibration T10-L5	2, 4 weeks of vibration	**•RT-qPCR**: ↑ Sox9 at 2 and 4 weeks	–	**•TUNEL:** ↑cell death	**•RT-qPCR:** ↑ ACAN and SOX9 at 2 and 4 weeks. ↑COL1 at 2 weeks. No difference in Col2a1	**•RT-qPCR**: No difference in MMP13, Adamts4, Adamts5 **IHC:** ↑ DIPEN and C1, 2C neoepitopes indicated aggrecan and collagen cleavage by MMPs in outer AF. No difference in NITEGE
	A mouse intervertebral disc degeneration modeled by surgically induced instability (69)	Surgical resection of posterior elements (facet joints, supra/interspinous ligaments) with no direct injury to the IVD	C57BL/6J	*N* = 3–11	Male	8 weeks	L4-L5	2, 4, 8, 12 weeks post-op	–	–	–	**•IHC:** ↑ in Col1, ColX with no change in Col2	•**IHC**: ↑ MMP13
	Development and characteri zation of a novel bipedal standing mouse model of intervertebral disc and facet joint degeneration (76)	Mice placed in 5 mm deep water containing spaces to induce bipedal standing posture (2x per day (6 h), 7 days a week). Control mice in water free space	C57BL/6J	*N* = 8	Male	8 weeks	Global but L3-L6 assessed	6, 10 weeks post induction	–	**•Histology** (H&E): ↓ size of NP cells	**•Histology** (H&E): ↓ # of cells	**•IHC**: ↓ Col2a1, Vimentin, Aggrecan	**•IHC:** ↑MMP13 and Osteocalcin
	A novel *in vivo* mouse intervertebral disc degeneration model induced by compressive suture (73)	Circumcision of tail skin followed by suture to exert excessive pressure on the IVD.	C57BL/6J	*N* = 3	Male	6–8 weeks	C8-C9	1 week pre-op 1, 2, 4 weeks post op	–	–	**•Histology** (H&E): ↓ # of cells	**•RT-qPCR:** ↑ Col1	**•WB:** ↑ MMP3 and TIMP 1 after 2 weeks **•RT-qPCR:** ↑ MMP3 and ↓ TIMP 1
**Puncture**	Injury-induced sequential transformation of notochordal nucleus pulposus to chondrogenic and fibrocartila ginous phenotype in the mouse (86)	Tail skin incised and IVD exposed. Puncture of IVD (31 G) to 1 mm depth into mice tail IVDs (noted as an “annular” puncture)	C57	*N* = 3–5	–	10–12 weeks	C4-C5 Injury with C3-C4 Control	Pre op and 1, 2, 6, 12 weeks post-op	**•RT-qPCR:** ↓ of sox9 over time.	**•Histology** (H&E, Safranin O): Notochordal Cells replaced by Chondrocyte-like cells in NP region	**•Histology** (H&E, Safranin O): ↓ Cell density	**•RT-qPCR:** ↓ACAN, Decorin, Vesican at 12 weeks overall. Temporal ↑ Col1 and ↓Col1 at 1 and 12 weeks. ↓col2a1 and sox9 over time. ↑ Col1a1 and fibronectin were indicative of fibrosis at 12 weeks.	
												**•*****in-situ*** **Hybridization:** ↓Collagen 2 and ↑ Collagen 1 in NP over time. ↓ Collagen 2 in AF over time **•IHC:** ↓Collagen 2 at 1, 2, and 6 weeks in AF. ↑fibronectin at 12 weeks with deposition of collagen 1 in NP region.	–
	Development of an *in vivo* mouse model of discogenic low back pain (84)	Ventral opening with Puncture of IVD to 0.7 mm with scalpel and followed removal of NP using micro scalpel	C57BL/6J	*N* = 3–19	Female	15 weeks	L4-L5, L5-L6, L6-S1	2,4,8,12 Weeks post-op	**•IHC:** ↑TNFα and IL-1B at 2 and 4 weeks	**•Histology** (Safranin O-Fast Green): Notochordal Cells replaced by Chondrocyte-like cells in NP region	–	–	–
**Genetic/Aging**	Accelerated aging of intervertebral discs in a mouse model of progeria (55)	Accelerated IVD degeneration in mouse model of Progeria due to ERCC1-XPF (DNA repair endonuclease deficiency)	ERCC1-XPF	*N* = 6	–	3, 20–23 week old ERCC1 Mice WT littermates (2–2.5 years old)	Global (Lumbar level assessed)	No interventions, assessed mice as is	–	–	**•TUNEL:** ↑ Apoptosis **•IHC:** P16INKa; ↑ senescence **•Histology** (H&E): ↓ cellularity, especially in CEP regions	**•IHC:** ↓ Aggrecan at 18–20 weeks compared to WT age control and similar to 2.5 yo WT mice •**RT-qPCR:** ↓ Versican and Aggrecan **•Histology** (Safranin O): ↓ Proteoglycan content	**•35S incorporation for PG synthesis**: 20-week Ercc1 incorporated less sulfate into protein than WT littermates. Errc1 have impaired PG synthesis -> contributes to reduced PG content.
												**•Mice induced DNA damage by mechlore** **thamine:** further induced proteoglycan loss in IVD and CEP	
	A novel mouse model of intervertebral disc degeneration shows altered cell fate and matrix homeostasis (56)	SM/J mice known for their poor regenerative abilities compared to super healer LG/J mice	SM/J	*N* = 6–10	Male Female	1, 4, 8 weeks, and 17 week	Global (Lumbar and Caudal IVDs assessed)	No interventions, assessed mice as is	**•IF:** ↓CA3, GLUT-1 (Slc2a1), and KRT-19. Also ↓ SDC4 (suggest lost of NP notochordal phenotype) **•RT-qPCR:** No differences in IL6, Adamts4, Fmod. ↓ in Vegfa (pro-survival factor for NP cells)	**•Histology** (SafraninO-Fast Green): loss of vacuolated cells with signs of hypertrophy **•RT-qPCR:** ↑Ctfg and Runx2 suggest hypertrophic chondrocyte phenotype of NP cells at 7 weeks.	**•TUNEL**: ↑cell death •Histology (SafraninO-Fast Green, Hematoxylin, PSR): ↓ NP cellularity, non-vacuolated cells)	**•IHC:** ↑ARGxx, indicating higher aggrecan turnover. **IF:** Strong aggrecan expression in NP but ↑ARGxx neoepitope at 17 weeks suggest ADAMTS-dependent degradation. ↑ Collagen X expression in NP at 17 weeks	**•IF:** ↑MMP13 and COLX in NP and AF at 17 weeks (also a marker of hypertrophic chondrocytes)

* *Key: “-” indicates assessment not specified. C, coccygeal levels; L, lumbar levels; T, Thoracic levels; WT, wild type mice; N, sample size used within the paper per group and assessment; ↓, decrease; ↑, increase; H&E, hematoxylin and Eosin staining; IHC, immunohistochemistry; RT-qPCR, Real Time quantitative polymerase chain reaction; WB, western blot; PSR, Picrosirius Red*.

**Table 2:** Comparison of mouse models and their tissue structural and functional assessments.

									**Tissue, Structural, and Functional Assessments**					
**Model type**	**Paper title (reference)**	**Model details**	**Strain**	**Sample size per assessment**	**Sex**	**Age**	**Levels affected or assessed**	**Timeline**	**Disc height index and hydration**	**Tissue level ecm content**	**Neuro vascular invasion**	**Disc morphology**	**Degeneration grade**	** Mechanics**
	Compression-induced degeneration of the intervertebral disc: an *in vivo* mouse model and finite-element study (99)	Compression device with pins through adjacent vertebrae with stress less of 0.4, 0.8 or 1.3 MPa or SHAM for 1 week and allowed to recuperate for 1 month	Swiss Webster mice	*N* = 4–7	Male	12 weeks	C9-C10	1 week and 1 month loading	–	–	–	**•Histology** (H&E, Safranin O): ↑ Disorder of AF region with increasing compression. 0.4 MPa loads regained AF organization, but 1.3 Mpa did not	–	**•**Neutral zone of the compressed disc was 33% greater than sham but bending stiffness and strength were not significantly different.
	Repeated exposure to high-frequency low-amplitude vibration induces degeneration of murine intervertebral discs and knee joints (78)	Vertical sinusoidal vibration (for mimicking humans whole-body vibration)–> 30 min per day, 5 days/week, 45 Hz with peak acceleration at 0.3 g	CD-1	N = 4–6	Male	10 weeks	Whole Body Vibration T10-L5	2, 4 weeks of vibration	–	**•Histology** (Safranin O-Fast Green &PSR): ↑GAG in interlameller matrix of AF	–	**•Histology** (Safranin O/fast green, Direct Red): No change at 2 weeks. At 4 weeks–> Loss of distinct NP/AF boundary, Collagen disorganization, focal disruptions in AF Lamellae and significant collagen disorganization with breaks in lamellae	**•Histology** (Safranin O/fast green, Direct Red): Modified Thomson grading scheme used –> ↑ AF Degeneration	–
**Mechanically Induced**	Migration of bone marrow-derived cells for endogenous repair in a new tail-looping disc degeneration model in the mouse: a pilot study (74)	BMCs derived from GFP transgenic mice and injected into the tail vein of WT mice. Tail Looping to induce IVDD–> Looping of C5-C13 with NP aspiration at C7-8, C8-9 (27G needle) as severely degenerate.	C57BL/6J	*N* = 9	Female	12 week old GFP donors. 8–10 week old BMC recipients	C2, C3 for control group, C10, C11 for mildly degenerate, and C7, C8 for severely degenerate	4,8,12 post op	–	–	–	**•Histology** (H&E, Safranin O): Loss of IVD height and wedging	**•Histology** (H&E, Safranin O): Nishimura, Mochida, and Nomura degeneration grading use –> significant wedging and degeneration in C7, C8 groups compared to C10, C11 and control groups	–
	A mouse intervertebral disc degeneration modeled by surgically induced instability (69)	Surgical resection of posterior elements (facet joints, supra/interspinous ligaments) with no direct injury to the IVD	C57BL/6J	*N* = 3–11	Male	8 weeks	L4-L5	2, 4, 8, 12 weeks post-op	**•Radiographs:** ↓DHI at 2 weeks with continuous decrease over time	**•Histology** (Safranin O): ↓Proteoglycan in NP and inner AF after 8 Weeks '–	–	**•Histology** (H&E, Safranin O): ↑Disorder of AF region followed ↑ NP degradation over time	**•Histology** (H&E, Safranin O): Masuda Histological scoring system used–> ↑ IVD Degeneration severity	–
	Development and Characterization of a Novel Bipedal Standing Mouse Model of Intervertebral Disc and Facet Joint Degeneration (76)	Mice placed in 5 mm deep water containing spaces to induce bipedal standing posture (2x per day (6 h), 7 days a week). Control mice in water free space	C57BL/6J	N = 8	Male	8 weeks	Global but L3-L6 assessed	6,10 weeks post induction	**•microCT:** ↓ DHI by 10 weeks	–	–	**•Histology** (H&E): Disorganized AF matrix, CEP height reduction.	**•Histology** (H&E): Used Smith histological grading scores –> ↑ IVD degeneration score	–
	A novel *in vivo* mouse intervertebral disc degeneration model induced by compressive suture (73)	Circumcision of tail skin followed by suture to exert excessive pressure on the IVD.	C57BL/6J	*N* = 3	Male	8 weeks	C8-C9	1 week pre-op 1, 2, 4 weeks post op	**•X-Ray:** ↓ DHI over time **•T2 MRI:** ↓ Hydration after 2 weeks	**•WB:** ↓ACAN, ↑COL1 in NP after 2 weeks	–	**•Histology** (H&E):↓ NP volume	–	**•**↑ IVD compressive stiffness over time
	A mouse model of lumbar spine instability (100)	Resection of L3-L5 spinous processes, supraspinous and interspinous ligaments	C57BL/6J	*N* = 8	–	8 weeks	L3-L5	1, 2, 8, 16 weeks post op	**•microCT:** ↓ IVD volume shown by 3D reconstruction starting at 2 weeks	–	–	**•microCT:** ↑ CEP volume and porosity, potentially indicating induced CEP hypertrophy. Bone loss at 16 weeks in vertebrae	**•Histology** (H&E and Safranin O): Used Boo histological grading Scores –> ↑ IVD degeneration score and ↑ CEP degeneration score	–
**Puncture**	Injury-induced sequential transformation of notochordal nucleus pulposus to chondrogenic and fibrocartilaginous phenotype in the mouse (86)	Tail skin incised and IVD exposed. Puncture of IVD (31 G) to 1 mm depth into mice tail IVDs (noted as an “annular” puncture)	C57	*N* = 3–5	–	10–12 weeks	C4-C5 Injury with C3-C4 Control	Pre op and 1,2,6,12 weeks post-op	**•Radiographs:** ↓DHI over time	**•DMMB/ Hoechst** (GAG/DNA): 30% ↓ in GAG at 6 weeks and 40% by 12 weeks.	–	**•Histology** (H&E, Safranin O): ↑Disorder of AF region (more serpentine) followed by ↑ NP degradation over time and clefts and indistinct NP-AF interface.	**•Histology** (H&E, Safranin O): Modified Masuda Histological scoring system used–> ↑ IVD Degeneration score over time	–
	Therapeutic effects of adenovirus-mediated growth and differentiation factor-5 in a mice disc degeneration model induced by annulus needle puncture (101)	Annular Puncture to L4, L5, L6 IVD with 27, 30 and 33 G needles. Ad-GDF5 or Ad-Luc were used on same mouse but different IVDs with 30 G Needle	Balb/c	*N* = 4 for model and *N* = 10 for gene therapy	–	8 weeks	L4-5, L5-6, L6-S1	1, 2, 4, 8 weeks post op	**•X-Ray** (1 and 2 weeks): ↓DHI 1 week post-injury but disc injected with ad-GDF 5 improved significantly by 2 weeks post-injection with no significant difference between treated and intact discs. **•T2 MRI (**1 and 2 weeks): In needle puncture all gauges ↓ hydration					
									by 2 weeks while both 27 g and 30 g showed loss at 1 week but not 33 g	**•DMMB/ Hoechst** (GAG/DNA): ↓ GAG in AD-Luc injection after 2 weeks but no decrease with Ad-GDF5 injection and treated groups had no difference with intact groups.	–	**•Histology** (H&E and Safranin O): Lost of AF structure at 8 weeks and more prominent in 27G. in treatment study, discs with ad-Luc or ad-GDF5 lost most of NP at first week with cells growing toward the center of the disc after ad-GDF5	–	–
	Needle puncture injury causes acute and long-term mechanical deficiency in a mouse model of intervertebral disc degeneration (79)	29 G (65% disc height) or 26 G (90% disc height) puncture to caudal IVDs through dorsal side	C57BL/6J (Retired Breeder Mice)	*N* = 2–5	–	7.5–9 months	C6-C7 and C8-C9	0, 8 weeks	–	**•DMMB** (GAG/wet weight): 26 G puncture showed ↓ in GAG compared to 29 G and controls at 8 weeks				
										**•Hydroxy proline** (Collagen/wet weight): 26 G puncture showed ↑ Collagen compared to 29 G and controls at 8 weeks	–	**Histology** (AB/PSR): 26 G induced disc collapses and AF disorganization at 8 weeks	–	**•** Normalized compressive stiffness, Torsional stiffness and torque range ↓ with needle gauge but not different between time points. Creep properties changed with needle size but not time. No differences in creep displacement
	Delayed onset of persistent discogenic axial and radiating pain after a single-level lumbar intervertebral disc injury in mice (85)	Ventral opening with Puncture of single level IVD *via* 30 G needle	CD-1	*N* = 8–15	Female	3 Months	L4/L5	0.5, 1.5, 3, 6, 9,10, 12 months post-op	**•X-Ray:** no change in DHI at 2 weeks with ↓ starting at 4 months **•MRI:** ↓ hydration in injured IVDs	**•Histology** (H&E, FAST): ↓ Proteoglycan	**•IHC and ir for PGP9.5:** innervation of outer AF in both injury and SHAM groups with ↑ innervation at 10 months.	**•Histology** (H&E, FAST): varying severity of building/herniation with reduced NP-AF compartmentalization	–	–
	Development of an *in vivo* mouse model of discogenic low back pain (84)	Ventral opening with Puncture of IVD to 0.7 mm with	C57BL/6J	*N* = 3–19	Female	15 weeks	L4-L5, L5-L6, L6-S1	2,4,8,12 Weeks post-op	**•microCT**: ↓ DHI over time	**•Histology** (Safranin O-Fast Green): ↓Proteoglycan in NP	**•IHC:** ↑ NGF at 2–12 weeks, ↑ PGP9.5 starting at	**•Histology** (Safranin O-Fast Green): ↑Disorder of AF	**•Histology** (Safranin O-Fast Green): Masuda Histological	–
		scalpel and followed removal of NP using micro scalpel								4 weeks	region and ↓ NP volume	scoring system used–> ↑ IVD Degeneration severity	
	Development of a Unique Mouse Intervertebral Disc Degeneration Model Using a Simple Novel Tool (102)	Subcutaneous Puncture of C4-5 IVD with 32 G (2 mm deep) Needle fixed to novel tool and confirmed by CT	C57BL/6J	*N* = 2–4	–	10 weeks	C4-C5	2,4,6 Weeks post-op	**•CT:** ↓ at 2 weeks normalized to control IVDs but ↑ at 4 to 6 weeks.	–	–	**•Histology** (H&E, Safranin O, Sirius Red): Distortion of NP and AF with AF collagen fiber disorientation	**•Histology** (H&E, Safranin O, Sirius Red): Masuda histology disc grade used –> ↑ IVD Degeneration in injured groups	–
	Lumbar intervertebral disc degeneration associated with axial and radiating low back pain in aging SPARC-null mice (54)	SARC-null mice (C57BL/6 ×129 SVJ background) –> SPARC decreased in humans with aging and degeneration	SPARC-null	*N* = 3–15	Male	6–78 weeks	Global (Lumber levels assessed)	No interventions, assessed mice as is	**•X-Ray:** ↓ DHI in SPARC-null mice at all time points compared to WT controls but both developed reduced DHI with age. DWI revealed wedging of IVD in aged mice of both strains but more severe in SPARC-null mice	–	–	**•Histology** (Multichrome FAST): ↑IVD Wedging. Loss of NP and AF compartmentalization by 24 weeks old. L3-L4 IVD abnormal in 100% of old SPARC-null mice with disc bulging, herniation, and spinal compression	**•Histology** (Multichrome FAST): ↑IVD Degeneration Severity after 24 weeks	–
**Genetic/Aging**	Accelerated Aging of Intervertebral Discs in a Mouse Model of Progeria (55)	Accelerated IVD degeneration in mouse model of Progeria due to ERCC1-XPF (DNA repair endonuclease deficiency)	ERCC1-XPF	N = 6	N/A	3 weeks and 20–23 week old ERCC1 Mice and WT littermates (2–2.5 years old)	Global (Lumbar level assessed)	No interventions, assessed mice as is	**•X-Ray or microCT:** ↓ DHI in ERCC1 mice at 3 weeks and 20 weeks comparable to old 2 yo WT mice. Image analysis also revealed kyphosis, increase bone porosity and loss of bone mineralization	**•DMMB/Pico Green** (GAG/DNA): ↓ GAG by 8 weeks with increase difference by 20 weeks.	–	–	–	–
	Increased innervation and sensory nervous system plasticity in a mouse model of low back pain due to intervertebral disc degeneration (103)	SPARC-null mice (C57BL/6 ×129 SVJ background) –> SPARC decreased in humans with aging and degeneration	SPARC-null	*N* = 5+	Male	1.5, 5–7, and 22–24 months	Global (Lumbar level assessed)	No interventions, assessed mice as is	–	–	**IHC:** for nerve fiber marker PGP9, calcitonin gene-related peptide CGRP in IVD to access innervation –> ↑nerve fiber ingrowth in SPARC-null old mice	–	–	–
	Behavioral signs of axial low back pain and motor impairment correlate with the severity of intervertebral disc degeneration in a mouse model (104)	SPARC-null mice (C57BL/6 × 129 SVJ background) –> SPARC decreased in humans with aging and degeneration	SPARC-null	*N* = 4–9	Female	6–78 weeks	Global (T1-S4 spine segments assessed)	No interventions, assessed mice as is	–	–	–	**•Histology** (Multichrome FAST): Reduced proteoglycan with NP and AF compartmental loss and AF lamellar disorganization	**•Histology** (Multichrome FAST): Using in house developed IVD degeneration severity scale –> increasing IVD degeneration with age. Same # of degenerate IVDs in SPARC-null and WT mice but SPARC-null IVDs had more severe degeneration	–
	A novel mouse model of intervertebral disc degeneration shows altered cell fate and matrix homeostasis (56)	SM/J mice known for their poor regenerative abilities compared to super healer LG/J mice	SM/J	*N* = 6–12	Male Female	1, 4, 8 and 17 weeks	Global (Lumbar and Caudal IVDs assessed)	No interventions, assessed mice as is	**•microCT:** ↓ Disc Height	**•FTIR**: ↑Collagen in NP region with no difference in inner/outer AF. ↓ Proteoglycan in NP with no changes in AF	–	**Histology** (SafraninO-Fast Green, Hematoxylin, PSR): ↓Disc Height, poor vertebral bone quality, Changed NP matrix composition, fibrous tissue invasion of NP, disorganized AF collagen infiltration of NP and indistinct CEP-NP and NP-AF interface	**•Histology** (SafraninO-Fast Green, Hematoxylin, PSR): Modified Thompson grading scale–> Degeneration at 8 weeks with increasing severity at 17 weeks indicative of early age degeneration.	**•**↑compressive Stiffness. Together with parameters of decreased height and poor vertebral bone quality, suggests compromised mechanical functionality
	Aging Of Mouse Intervertebral Disc And Association With Back Pain (50)	Naturally aged mice	C57BL/6J Mice or FVB	*N* = 5–13	Male Female	3–24 months	Global (Lumbar level assessed)	No interventions, assessed mice as is	**•X-Ray absorptiometry (DEXA):** ↓ DHI in 2 yo mice. BMD ↓ with age in both sexes.	–	**•IHC:** ↑ vascular (CD31) and neuronal invasion (PGP9.5) into IVD due to aging	**•Histology** (H&E): 3 months (young) have uniform IVD structure with NP and AF distinct, 1 year (mid aged adult) AF less structurally intact and NP diffused, 2 year (old) diverse phenotype and hypo cellularity. 2 yo mice and shorter and wider	–	–

* *Key: “- ” indicates assessment not specified. C, coccygeal levels; L, lumbar levels; T, Thoracic levels; WT, wild type mice; N, sample size used within the paper per group and assessment; ↓, decrease; ↑, increase; H&E, hematoxylin and Eosin staining; IHC, immunohistochemistry; RT-qPCR, Real Time quantitative polymerase chain reaction; WB, western blot; PSR, Picrosirius Red; microCT, micro computed tomography; MRI, magnetic resonance imaging; FTIR, Fourier-transform infrared spectroscopy; DMMB, dimethylmethylene blue assay*.

**Table 3:** Comparison of mouse models and their pain behavioral assessments.

										**Pain behavioral assessments**						
**Model type**	**Paper title (reference)**	**Model details**	**Strain**	**Sample size per assessment**	**Sex**	**Age**	**Levels affected or assessed**	**Timeline**	***In vitro*** **neuronal assess ments**	**Spont aneous pain**	**Locomotor**	**Mechani cal sensitivity**	**Cold sensitivity**	**Heat sensitivity**	**Axial discomfort**	**Lateral flexion**
**Puncture**	Delayed onset of persistent discogenic axial and radiating pain after a single-level lumbar intervertebral disc injury in mice (85)	Ventral opening with Puncture of single level IVD *via* 30G needle	CD-1	*N* = 8–15	Female	3 Months	L4/L5	0.5, 1.5, 3, 6, 9,10, 12 months post-op	–	–	**•Rotarod:** **↑** locomotor capacity in injury mice at 1.5 month but no change otherwise	**•von-Frey**:No Difference across time	**•Acetone:** ↑ sensitivity at 2 weeks and 12 months	–	**•Grip Force:** no change **•Tail suspension:** delayed onset avoidance of axial stretch at 3–9 months post injury	–
	Development of an *in vivo* mouse model of discogenic low back pain (84)	Ventral opening with Puncture of IVD to 0.7 mm with scalpel and followed removal of NP using micro scalpel	C57BL/6J	*N* = 3–19	Female	15 weeks	L4-L5, L5-L6, L6-S1	2,4,8,12 Weeks post-op	**•IHC on DRG:** ↑ CGRP, TrKA, NGF across all time points **RT-qPCR on DRG:** ↑ CGRP, NGF, SubstanceP, TrKA, BDNF, TRPV1, NPY, Nav1.7, Nav1.8 **•IHC on Spinal Cord:** ↑GFAP astrocyte, ↑Microglia (IBA-1) at 2 weeks but decreased after 4 weeks in dorsal horn. Correlated with IVD inflammation	**•Burrowing:** ↓ burrowing after 4 weeks, indicates chronic pain **•Open Field:** ↓ rearing at 12 weeks with no difference in ambulation activity. Suggest vertical motion deficit but no deficit in horizontal motion.	–	**•von-Frey** (hind Paw): ↓ Threshold.	**•Acetone:** ↑time spent in evoked behavior by 8 and 12 weeks. Suggests cold hyperalgesia	**•Hot Plate:** ↓Latency by 8 and 12 weeks. Suggests cold hyperalgesia	–	–
**Genetic/Aging**	Behavioral signs of chronic back pain in the SPARC-null mouse (105)	SPARC-null mice (C57BL/6 × 129SVJ background) –> SPARC decreased in humans with aging and degeneration	SPARC-null	N = 5–10	Male	Young (3 months) Old (9 month) (6 and 9 month old controls due to mice availability)	Global	No interventions, assessed mice as is.	–	–	**•Rotarod:** No difference between groups. Suggests no generalized nervous system dysfunction	**•von-Frey** (hind Paw and Lower Back): No Difference in strain or age	**•Acetone** (hind paw & lower back): No difference between strain in young mice. ↑ Cold evoked behaviors in old mice in hind paw and back. ↑ cold sensitivity with increasing age in SPARC-null mice **•Tail Immersion**: No difference	•**Hargre aves (**hind paw**):** No difference between stain in young or old mice	**•Grip Force:** ↓ resistive force **•Tail suspension:** ↓ time immobile with ↑ rearing/self-supported time	–
	Lumbar intervertebral disc degeneration associated with axial and radiating low back pain in aging SPARC-null mice (54)	SPARC -null mice (C57BL/6 × 129SVJ background) –> SPARC decreased in humans with aging and degeneration	SPARC-null	*N* = 3–15	Male	6–78 weeks	Global (Lumbar level assessed)	No interventions, assessed mice as is.	–	**•Open Field**: Reduced voluntary activity in SPARC-null mice	**•Rotarod:** **↑** Improvement with age in SPARC-Null and WT mice but decrease at >70 weeks in SPARC-null while WT mice plateaued. Potentially due to learned behavior/practice being on platform	**•von-Frey** (hind Paw & Lower Back): No Difference in plantar paw but slightly ↑ sensitivity in low back	**•Acetone** (hind paw & lower back): ↑ Cold sensitivity compared to WT and WT also showed signs of ↑ cold sensitivity by 1 year old in paw. No change in low back between strain **•Tail Immersion:** No difference **•Cold water paw immersion**: SPARC- null hypersensitivity as early as 8–16 weeks old while aging WT exhibited this sensitivity during 2nd year of life.	**•Hargre aves:** No difference **•Tail Flick:** No difference	**•Grip Force:** ↓ resistive force with age in SPARC mice while WT got stronger with age **•Tail suspen sion:** ↓ time immobile with ↑ rearing/self supported time. 2nd year showed ↑ immobility time and decreased time in self support.	**•Flex Maze:** SPARC-null mice demonstrated signs of activity-induced decreases in physical function associated with lateral flexion and spontaneously reduce their exploration speed. (Assay developed in Stone Lab)
	Increased innervation and sensory nervous system plasticity in a mouse model of low back pain due to intervertebral disc degeneration (103)	SPARC -null mice (C57BL/6 × 129SVJ background) –> SPARC decreased in humans with aging and degeneration	SPARC-null	*N* = 5+	Male	1.5 months (young), 6–7 months (middle age), and 22–24 months (old)	Global (Lumbar level assessed)	No interventions, assessed mice as is.	**•Immunore activity on DRGs and Spinal cord (ir**): ↑CGRP and NPY neurons correlate with age and strain. n spinal cord, effect of age on astrocyte and microglial expression with a weak effect of strain and effect on GFAP (astrocytes). ↑CGRP in lower lumbar spinal cord when compared to upper lumbar spinal cord	–	**•Rotarod:** ↓physical function significantly with age in both mice with no effect of genotype.	**•von-Frey** (hind Paw & Lower Back): No Difference across time	**•Acetone:** ↑hypersensitivity with young and middle aged SPARC-null mice similar to old WT mice **•Cold Plate:** ↑hypersensitivity	**•Hargre aves**: No effect of age or strain	**•Grip Force:** hypersensitivity/ impairment in grip test **•Tail suspension:** decrease immobile time	–
	Behavioral signs of axial low back pain and motor impairment correlate with the severity of intervertebral disc degeneration in a mouse model (104)	SPARC -null mice (C57BL/6 × 129SVJ background) –> SPARC decreased in humans with aging and degeneration	SPARC-null	*N* = 4–9	Female	6–78 weeks	Global (T1-S4 spine segments assessed)	No interventions, assessed mice as is	–	–	**•Rotarod:** motor impairment >18 weeks correlated with upper lumbar IVD degeneration (L1-L2, L2-L3) SPARC-null mice.	**•von-Frey** (hind Paw & Lower Back): No Difference across time	**•Acetone:** cold hypersensitivity at > 18 weeks with no correlation between hypersensitivity in hind paw to IVDD	–	**•Grip Force:** ↓ tolerance to axial stretching and correlated with degeneration score of lower lumbar levels (L3-L6)	–
	Aging of mouse intervertebral disc and association with back pain (50)	Naturally aged mice	C57BL/6J Mice or FVB	N = 5–13	Male Female	2–24 months	Global (Lumbar level assessed)	No interventions, assessed mice as is	**•IHC (DRGs)**: ↑ Nav1.8, Nav 1.9, which correspond to reduced pain threshold in mice	**•Open Field:** ↓ rearing (time spend on hind limbs) with age as it requires axial stretch, trunk stability, and lower body strength. Older mice preferred to explore on 4 limbs	–	–	**Acetone:** ↑ Response with age suggestive of cold allodynia. Further supported by ↑ TRPA1 (noxious cold channels) with age	**•Capsai cin:** used to measure thermal hyperalgesia in this study but found no correlation with age, sex or weight	**•Tail Suspension:** ↑ Rearing activity in older mice to alleviate discomfort due to axial stretching	–

* *Key: “-” indicates assessment not specified. L, lumbar levels; T, Thoracic levels; WT, wild type mice; N, sample size used within the paper per group and assessment; ↓, decrease; ↑, increase; IHC, immunohistochemistry; ir, immunoreactivity; RT-qPCR, Real Time quantitative polymerase chain reaction*.

## Molecular and Cellular Assessments

Several molecular and cellular assessment methods can be used to characterize IVD degeneration. Transcriptome expression utilizing quantitative reverse transcription-polymerase chain reaction (RT-qPCR) can be used for the evaluation of healthy phenotypic markers, matrix markers, catabolic enzymes [i.e., matrix metalloproteinase (MMPs), ADAM Metallopeptidase (ADAMTs), Tissue inhibitor matrix metalloproteinase 1 (TIMP1)] and inflammatory cytokines (55, 56, 73, 86, 106). However, mice IVDs are small in size, reducing the ability to obtain sufficient amounts of total RNA per disc. Thus, the pooling of several IVD levels is often required, limiting the ability to assess level effects within each mouse. *In-situ* hybridization can also be used to localize and quantify transcriptome expression (86, 99). Commonly, immunohistochemistry (IHC) and immunofluorescence (IF) are used to detect target proteins within cells of histological tissue sections. This includes cellular expression of matrix proteins such as collagen, aggrecan, neoepitopes (DIPEN for aggrecan cleavage, C1, 2C for collagen 1 and 2 cleavage), fibronectin, or MMPs, as shown in Table 1.

To quantify apoptotic cells within IVD tissue, the TUNEL assay can be used. Cellularity within the different IVD compartments is commonly characterized *via* histological stains, namely Hematoxylin & Eosin (H&E), Safranin O-with or without Fast Green, or FAST multi-chrome staining. Additionally, the loss of a notochordal phenotype, transition to a mature NP phenotype, or cell clustering/hypertrophy can be accessed *via* changes in cell morphology in combination with phenotypic markers either *via* gene or protein measures (107). In addition to IVD cells, immune cells play a large role in the pathogenesis of LBP including macrophages and mast cell infiltration which can be accessed *via* IHC/IF, green fluorescent protein labeled transgenic mice (32–34, 36).

### Comparisons of Molecular and Cellular Assessments Between Models

Most mouse models that included molecular and cellular assessments described in this section have based their assessment of disc degeneration on changes in cellularity and ECM markers, with few papers examining changes in phenotypic and inflammatory markers (Table 1). This may be due to the limited size of mice IVDs and availability of tissue for multiple assessments as previously mentioned. Multiple strains of mice have been used including wild-type, CD-1, SPARC-null, SM/J, and Swiss Webster mice (55, 56, 69, 73, 76, 78, 84, 86, 99); interestingly only one study compared both male and female mice in this section (56). Parameters have been evaluated at different time points ranging from pre-op (if mechanically/puncture induced), 0 days (immediately after puncture) and up to 2 years. These cell/molecular level comparisons can be categorized into cell morphology & cellularity, cell-specific Extracellular Matrix Genes, and phenotypic & inflammatory markers. Despite immune infiltration as a contributor to LBP, only expression of inflammatory cytokines was assessed in the following studies.

#### Cell Morphology and Cellularity

In all studies, regardless of the induction method, mice strain, or sex, the intervention to induce degeneration increased apoptosis and decreased cellularity in NP and CEP regions of the IVD (55, 56, 73, 76, 78, 86, 99). Interestingly, in the bipedal mouse, the size of NP cells decreased compared to control mice whereas, in the SM/J mice, cellular hypertrophy was observed with loss of vacuolated cells (56, 76). This loss of notochordal cell phenotype and transition to chondrocyte-like mature NP cells was also observed in compression, and puncture models of lumbar and caudal IVDs (84, 86, 99).

#### Extracellular Matrix Genes

As maintenance of ECM homeostasis is critical for IVD structure/function, it is not surprising that most models include assessments of cellular ECM. MMP13 expression was typically assessed in mechanically induced models and found to be upregulated as early as 1-week post-induction. Interestingly, the whole-body vibration model showed no differences in MMP13 or ADAMTs4/5 compared to other mechanically induced models (78). Most studies demonstrated decreased collagen 2 and aggrecan expression in the IVD while collagen 1 expression was inconsistent across models, time points within models, as well as assessment method (i.e., RT-qPCR on entire IVD or IHC); suggesting different degenerative mechanisms between different models (73, 78, 86, 99).

#### Phenotypic and Inflammatory Markers

The whole-body vibration model by McCann et al. which uses CD-1 mice demonstrated increased SRY-Box Transcription Factor 9 (SOX9) gene expression at 2 and 4 weeks while puncture of the caudal IVD by Yang et al. in a C57 mouse showed decreased SOX9 over time after 12 weeks (78, 86). The SM/J mouse model which pooled IVDs for transcriptome analysis found decreased expression of keratin-19 (KRT-19), CA3, Syndecan 4 (SDC4), and Glucose transport 1 (GLUT-1) with increased expression of connective tissue growth factor (CTGF) and RUNX2, suggesting loss of a healthy NP phenotype (56). When assessing inflammatory cytokines in wild-type mice with punctured lumbar IVDs increased TNFα and IL-1β expression was observed at 2 and 4 weeks, indicative of acute inflammation (84). Interestingly, in SM/J mice no differences in IL-6, ADAMTs4, or fibromodulin (FMOD) were identified, but a decrease in vascular endothelial growth factor (VEGFα) gene expression was observed (56, 108), suggesting differences in both phenotypic and inflammatory marker expression across models.

### Translatability of Model Results to the Human Condition

Molecular and cellular changes that characterize IVD degeneration in humans include decreased cellularity (with increased apoptosis and senescence), immune infiltration, loss of proteoglycan/GAG & collagen 2, and increased fibrosis/collagen 1 in the NP (109). Many of the mouse models of IVD degeneration reviewed here demonstrated similar molecular and cellular changes as the human. However, one major difference was inconsistent changes in collagen 1 across different studies. In humans, large vacuolated notochordal cells predominate before 10 years of age and transition into chondrocyte-like cells during adolescence. In degeneration, the NP decreases in cellularity with more degenerate cells (110). This characteristic change in cell phenotype and number was observed in puncture models along with the SM/J model, providing clinical relevance to the human condition on the cellular level for using these models to study IVD degeneration and regenerative therapies. Healthy NP phenotypic markers identified in the human IVD include GLUT-1, Aggrecan/collagen 2 ratios>20, sonic hedgehog, Brachyury, KRT18/19, CA12, and CD24 (111). Markers such as GLUT-1, aggrecan, KRT-19 were also decreased in mice with IVD degeneration. Unlike the NP, the AF and CEP are under characterized and some studies have suggested COL1A1, Elastin (ELN), Mohawk (MKX), and Scleraxis (SCX) as potential markers of the healthy AF (112–115). In addition, there is an increase in catabolic and pro-inflammatory markers (i.e., TNFα, IL-1β, IL-6, MMPs, ADAMTs) in degenerate human discs compared to healthy IVDs (116, 117). Increased expression of TNFα, MMP13, and IL-1β in mice IVDs was consistent with the human condition while many mouse models found no differences ADAMTs genes expression. Importantly, infiltration of immune cells, notably macrophages and mast cells, have been found in degenerate human IVDs and mouse IVDs post-injury, warranting the need to assess immune cell infiltration in mice models to account for immune-related systemic effects (32–34, 36, 37).

### Critical Molecular and Cellular Assessments for Mouse Models of IVD Degeneration

Given the molecular & cellular parameters highlighted here, gene expression seemed to vary between studies and was often inconsistent when comparing relevant human markers while histological and IHC assessments for protein level assessments were more consistent when compared with the human condition. Thus, in terms of molecular markers, TNFα and IL-1β are critical inflammatory markers while MMP13 may be a good catabolic marker for assessment of degeneration in mice IVDs as they are most relevant to the human condition. However, due to size limitations, the temporal expression on the gene level, and difficulty isolating high-quality RNA from single-level IVDs, transcriptome level changes may be more challenging to corroborate between mouse models. Histological stains such as H&E, Safranin O, are good for the assessment of cellularity while TUNEL assay can give information related to apoptosis consistent with human IVD degeneration.

## Tissue and Structural/Functional Assessments

Assays such as dimethyl methylene blue (DMMB) are used to measure GAG and are typically normalized to DNA content (Hoechst or Pico green) or tissue weight (55, 79, 86, 101). For collagen content, Hydroxyproline or Sircol assays are used, although the specific collagen type is indistinguishable. Structural tissue level assessments are commonly assessed *via* histological staining such as H&E for overall tissue organization, Safranin O (with/without Fast green) or alcian blue (AB) for proteoglycans, picrosirius red (PSR) or Elastica van Gieson for collagen, Azan trichrome and Goldner's Masson's for more general cartilage and bone, and Weigert's Resorcin Fuchsin for elastin (50, 73, 82, 102, 118–121). Multichromatic staining (i.e., FAST) can enhance visualization of tissue-level changes (122, 123). Histological grading schemes can be used to determine the degree or severity of IVD degeneration (82, 102, 123).

In addition to histology, *in vivo* and *ex vivo* imaging methods including, radiographs, micro-computed tomography (microCT), and magnetic resonance imaging (MRI) are widely used for characterizing gross characteristics of tissue integrity/structure such as tissue hydration, disc height, grade of degeneration, and bone mineral density (50, 54–56, 69, 73, 76, 84, 86, 101, 102). DHI and Disc wedging index (DWI) can be derived from image analyses where DHI is the thickness/height of the IVD relative to vertebrae length and DWI is a measure of the anterior vs. posterior angulation, or wedging, in the sagittal plane (88, 124). For example, a DWI > 1 indicates that the IVD is wedged with pressure at the posterior region and increased likelihood of pressure to the spinal cord and dorsal root ganglion (DRGs). In addition, Pfirrmann grading, an MRI based degeneration scale for humans ranging from 1 = healthy to 5 = severe disease, can be determined *via* calculating MRI index and normalization to both control IVDs and background signal intensity as described in Onishi et al. (82, 125).

The mechanical behaviors of mice motion segments have been evaluated both *in vitro* or *in vivo* and mechanical testing is often used to inform or quantify IVD structure-function (99, 126, 127). Multiple testing protocols have been developed to assess behaviors *in vitro* under multiple loading modes such as compression/tension, torsion, and creep using a mechanical testing system (Table 2). Depending on the mode of testing, mechanical parameters can be extracted either directly from the raw data or derived from fitting the data with various mathematical models and used to quantify changes that occur with degeneration.

### Comparisons of Structural and Functional Assessments Between Models

In mouse models of IVD degeneration (Table 2), most studies assessed changes in disc structure & degeneration grade *via* histology, and DHI *via* radiographs or microCT. Interestingly, despite being critical parameters of disc structure/function, few studies assessed mechanics or disc hydration. Multiple genetic strains of mice were used, including the wild-type, CD-1, SPARC-null, SM/J, ERCC1-XPF, and Swiss Webster mice with only 2 studies comparing both male and female mice. Parameters were assessed from pre-op to 2 years of age and included imaging parameters (X-ray, microCT, MRI) for DHI and hydration, histology for disc structure and grade, neurovascular invasion, and mechanics.

#### Imaging: Radiographs, MicroCT, and MRI

Radiographs and microCT imaging are commonly used across models to assess DHI. Most models demonstrated decreased DHI in degenerative groups except for the subcutaneous puncture model where DHI initially decreased at 2 weeks but *increased* at 4 to 6-week time points. Mice with lumbar instability demonstrated increased CEP volume and porosity in mice along with decreased IVD volume starting at 2 weeks and bone loss at 16 weeks (100). Genetic SPARC-null mice assessed DWI and found wedging of IVDs in both SPARC-null and old wild-type mice, but with increased severity in SPARC-null groups (54). Similarly, ERCC1 mice demonstrated decreased DHI at 3 weeks which was similar to old aged mice with decreased bone mineralization (50, 55). In both mouse tail compression and needle puncture models, IVD hydration decreased as early as 2 weeks post-op and even earlier in larger needle puncture models (73, 101). Although MRI measurements were included, Pfirrmann grades were not assessed in these studies as per Onishi et al. (82).

#### Histological Assessment, ECM, and Grading

Histological staining of mice spines or motion segments has been widely used to assess changes in IVD structure. All models showed reduced proteoglycan in the NP, AF disorganization with serpentine-like lamellae, decreased IVD height, and reduced distinction of NP and AF boundaries indicative of collagen infiltration into the NP region. This change in structure correlates with decreased GAG content in all models of degeneration in the NP region and increases in collagen 1. However, these changes occurred at different time points depending on the degree of puncture (i.e., increased needle size showed degeneration early on), compression (increasing AF disorganization with increased compression), and mode of mechanical disruption (direct injury to IVD vs. indirect mechanic induction such as in bipedal mice) (78, 86, 99, 128). Compared to wild-type mice that often show signs of IVD degeneration at 1 year, mechanically induced/puncture models exhibit characteristics of degeneration at earlier times (~2 weeks) and genetic models (SPARC-null, SM/J, and ERCC1 mice) show signs at ~3 weeks (50, 54–56). As expected, IVD grades increased with the severity of degeneration across all models but the grading scheme varied across studies (Table 2). Thus, a standardized histopathology scoring system using machine learning algorithms for the mouse model as well as the human has been proposed (129, 130).

#### Neurovascular Invasion

Lumbar IVD puncture models demonstrate increased nerve growth factor (NGF) expression starting at 2 weeks and Protein Gene Product 9.5 (PGP9.5) starting at 4 weeks similar to old wild-type mice, with increased expression of PGP9.5 and vascular marker CD31 with age (50, 84). In SPARC-null mice, PGP9.5 and calcitonin gene-related peptide (CGRP) expression were both upregulated in IVDs, indicative of nerve fiber ingrowth in these different models regardless of the mechanism of IVD degeneration (103).

#### IVD Mechanics

One of the IVD's main functions in the spine is to facilitate motion while supporting physiologic loads and the objective of many therapies is to restore the mechanical function of the IVD. Thus, assessing the mechanical behaviors and properties of the IVD is critical to characterize disease progression and assess therapeutic efficacy. However, the small-scale mechanical testing of mice motion segments often requires custom devices and has not been universally applied. The studies that have conducted mechanical testing on mouse degeneration models have demonstrated that degeneration induced *via* compressive overload on caudal IVDs resulted in increased compressive stiffness with no difference in bending stiffness or strength compared to healthy controls (99). However, under creep loading degenerated IVDs from the same model demonstrated a reduction in the strain-dependence of swelling pressure determined from fitting with a fluid transport model (131). Another model of compressive force induced *via* suture showed increased compressive stiffness over 4 weeks (73). In caudal puncture models, compressive stiffness, torsional stiffness, and torque all increased with needle size (79). SM/J mice showed increased compressive stiffness, suggesting that the mechanical function was altered in these models compared to healthy controls (56).

### Translatability of Model Results to the Human Condition

On the structural level, human IVD degeneration is characterized by dehydration of the NP due to proteoglycan loss, AF disorganization resulting in a loss of AF-NP boundaries, decreased DHI, and sclerosis of the CEP. In the case of herniated IVDs, the IVD contains AF fissures leading to NP protrusion (109, 117, 132, 133). These changes in the NP and AF were all observed in the mice models discussed here however degenerative changes in the CEP region were mostly unquantified (Table 2). Additionally, this loss of IVD architecture in degeneration creates a permissive environment for vasculature and nerve ingrowth into the usually avascular and aneural healthy IVD which may be a source of pain in DBP (134–136). This correlates with the increased nerve fiber ingrowth in degenerative mice IVD as seen in the assessed studies. In the clinic, structural changes in the IVDs of living patients are often detected *via* MRI and CT or x-ray which correlates with the use of MRI and microCT in mouse models to detect structural changes in the IVD, spinal cord, and adjacent tissues. Furthermore, human IVDs can also be assessed macroscopically *ex vivo* using the Thompson grading scheme (137). However, this grading scheme is limited clinically as it is typically performed on cadaveric tissue. Instead, the Pfirrmann grading scale can be used to grade human IVDs imaged *via* T2-weighted MRI assessing DHI, hydration, and NP/AF compartmentalization (125). The present mouse studies primarily utilized histological assessments which may be attributed to the accessibility of histological techniques compared to imaging modalities such as MRI. It is also important to note that degenerate IVDs imaged as black discs on MRI do not always correlate with LBP; patients can have several degenerate IVDs and experience little or no pain (95, 138). This highlights a need to also quantify measures of pain as changes on the structural level alone cannot always be used as a proxy for diagnosis or treatment of LBP.

These structural changes that develop during degeneration induce corresponding changes in IVD mechanical behaviors & properties. Mechanical properties of tissues can vary substantially based on the size and dimensions of the tested material; similar to how a thick piece of rope is stiffer than a thin piece of rope of the same material. This concept also applies to the IVD as the physical dimensions and associated axial and torsional properties vary between species due to IVD size (38, 139, 140). To account for these geometric differences the properties can be normalized to the geometry of the specimen, similar to material properties in material science, and thereby allow comparison of properties independent of geometric biases. For example, if the mechanical behaviors of IVDs of various species and anatomical locations (i.e., lumbar and caudal) are normalized to their respective geometries, the differences between species and region is reduced and in some cases are insignificant which allows direct comparison between mice and human IVDs (140, 141). For example, torsional stiffness (K) and torque range (TR) are often used to characterize the rotational behaviors of IVDs. Comparing the raw measurements there is a difference of ~4 orders of magnitude between human (K_Human_ = 3.18 ± 0.89 Nm/degree) and mouse (K_Mouse_ = 1.1^*^10^−4^ ± 1.83^*^10^−5^ Nm/degree) IVDs. However, after normalization to the disc height and polar moment of inertia (i.e., the shape's resistance to torsional deformation) there are no significant differences between the two species (139). Studies have further compared the normalized axial and viscoelastic creep properties across species and demonstrated that normalized material properties are largely conserved across species (139, 140, 142). This suggests that when comparing normalized mechanical parameters, mouse models are reasonable models of the human condition. In addition to comparing normalized mechanical parameters extracted directly from mechanical testing, other studies have compared how analytically derived parameters determined from fitting mechanical data with theoretical models under similar loading conditions compare between regions and with degeneration. For example, the response of human and mouse IVDs to creep loading has been fitted with a fluid-transport model, and similar decreases in the parameter corresponding to the strain dependence of swelling pressure decreases in both mouse models and degenerated human IVDs (77, 99, 143).

### Critical Structural and Functional Assessments for Mouse Models of IVD Degeneration

For the structural and functional parameters assessed, histological staining was consistently used across most mouse models with limited differences between models and correlated well with changes observed in human IVD degeneration. Thus, histological assessment of reduced proteoglycan in the NP, reduced NP/AF demarcation, and disorganization of the AF matrix are all critical factors to assess when confirming degeneration in mouse models and the regenerative potential of therapies. DMMB assessments are consistent between all studies not only within these models but across the IVD field. Collectively, the combination of these observations in addition to standardized histopathological scoring systems will improve the comparability across studies and their translation to the human condition (129, 130). T2-weighted MRI to assess Pfirrmann grade, IVD hydration, and microCT for DHI is also strongly recommended as these assessments are used clinically in human patients and parameters are consistent across mouse models. Notability, assessment of mechanical stiffness is important given the consistent increase in compressive stiffness observed in diseased mice and human IVDs, yet the expertise and access to equipment to do these tests may warrant collaborations across disciplines.

## Pain Behavioral Assessments

While there is a clear correlation between the severity of IVD degeneration and LBP, not all clinical cases of IVD degeneration present with painful symptoms in humans (144). Thus, assessments of pain are crucial in the evaluation of therapies aiming to treat symptomatic pain in addition to *ex vivo* IVD measures. While some pain markers can be evaluated *ex vivo*, pain perception is a cortical activity requiring the peripheral and central nervous systems (145). Therefore, the brain is required to perceive pain, and this is a component that *in vitro* models cannot recapitulate as pain is due to the complex interplay between the different components of the nervous system and surrounding tissue. The following sections will summarize the different classifications of pain associated with LBP as well as the available behavioral assessments for mice models categorized into evoked vs. spontaneous pain measures. Based on these comparisons, recommended assessments will be discussed.

### Types of Pain and Changes in the Sensory Nervous System

Pain can be categorized broadly into neuropathic or nociceptive pain and LBP likely has neuropathic and nociceptive contributors. Neuropathic pain results from direct injury to neuronal tissue. In the case of LBP, the pain can arise from IVD herniation which causes pressure on the nerve root and innervating DRGs, resulting in inflammation, radiculopathy, and damage to the nervous system directly. Nociceptive pain results from injury to non-neuronal tissues such as from the muscles surrounding the IVD or the IVD joint itself, where neoinnervation can occur and peripheral nociceptive neurons are stimulated/excited and transmit signals to the central nervous system (146, 147). Although not widely understood, an emerging third type of pain known as “nociplastic pain” may also play a role in LBP with a distinct mechanism unlike neuropathic or nociceptive pain (148). LBP can also be classified by the duration of pain (acute or chronic). Acute LBP occurs due to tissue trauma with patient recovery within a month, is typically self-limiting, and becomes subacute in the 1–3 month range (149). Chronic LBP lasts more than 12 weeks and patients who present with acute pain may develop chronic LBP over time (150, 151). Acute pain is often protective and alerts individuals to potentially damaging environmental stimuli, thereby aiding in the healing process. Meanwhile, chronic pain serves no protective role and can be debilitating (152). LBP can also be classified into spontaneous or movement evoked discomfort with localization to the lower back and spine, or, radiating pain which also affects the legs due to injury or inflammation of the nerve root (153, 154).

Anatomically, the IVD is innervated bilaterally by neurons with cell bodies residing in the DRGs. DRGs are heterogeneous (including proprioceptors, nociceptors, Schwann cells, fibroblasts and satellite glial cells) and are responsible for transmitting signals from the periphery to the central nervous system *via* projections into the dorsal horn (155, 156). Functional changes and sensitization of the sensory neurons due to IVD degeneration can lead to neuropathic or nociceptive pain *via* multiple mechanisms (147). During IVD degeneration, nerve endings expand from the outer AF to the inner AF and NP regions of the disc due to decreases in chondroitin-sulfated proteoglycans and elevated levels of neurotrophic factors [i.e., neurotrophins such as NGF and brain-derived neurotrophic factor (BDNF)] and this can lead to nerve sensitization (29, 136, 147, 157–160). In addition, the degenerate IVD is largely innervated by small nociceptors that express voltage-gated sodium channels (VGSCs) or transient receptor potential cation channel subfamily V member 1 **(**TRPV1) that regulate neuronal activity (161). Persistent inflammation in the IVD can sensitize these neurons and induce changes, causing altered action potential duration, hyper-excitability, lowered thresholds to stimuli, and enhanced pain as observed in rodent models (50, 161–163). The activation of Protease-activated receptor 2 (PAR2) on DRG sensory neurons can regulate acute and chronic pain by activating the extracellular signal-regulated protein kinase (ERK 1/2) signaling pathway (162, 164, 165). However, the exact mechanisms driving LBP remain unclear with a limited number of mice models investigating changes at the DRG level.

### *In vitro* Pain Assessments

*In vitro* pain assessments in this section are related to neuronal function/activity of innervating DRGs and spinal cord taken from mice models *ex vivo* while neurovascular invasion (nerve ingrowth and neo-angiogenesis) into IVD tissue were addressed in the previous section. *Ex vivo* characterization of isolated neurons has been performed to determine the role of DRGs in discogenic neuropathic/nociceptive pain. Electrophysiology and IHC have provided insight into the function and expression patterns of the ion channels/receptors and have highlighted their role in the pain signaling pathway in rat models but few ion/channels have been assessed in mice (161, 166). In addition, calcium imaging is a standard method to quantify changes in ion channel activity in the neurons (167). At the gene level, transcriptome analysis of the DRG neurons has been used to identify key genes associated with pain perception (168). In addition, IHC on the DRGs from a lumbar IVD puncture model demonstrated increased key pain markers, CGRP, Tropomyosin receptor kinase A (TrKA), and NGF from 2 to 12 weeks post-op and this is further supported by increased gene expression of CGRP, substance P, TrKA, BDNF, TRPV1, Neuropeptide Y (NPY), VGSCs Nav1.7/1.8 (84). In SPARC-null mice, CGRP reactivity was also elevated in addition to NPY in DRG neurons (103). IHC on the spinal cord showed increased expression of astrocyte marker, Glial fibrillary acidic protein (GFAP), and also microglia expression at 2 weeks in the puncture model, potentially due to inflammation from injury, while SPARC-null mice found increasing numbers of astrocyte and microglial cells with age (84, 169). Thus, the DRGs are key determinants in the induction and maintenance of both neuropathic and nociceptive pain, making their assessment in LBP models critical.

### *In vivo* Pain Behavior Assessment

**Figure 2:**
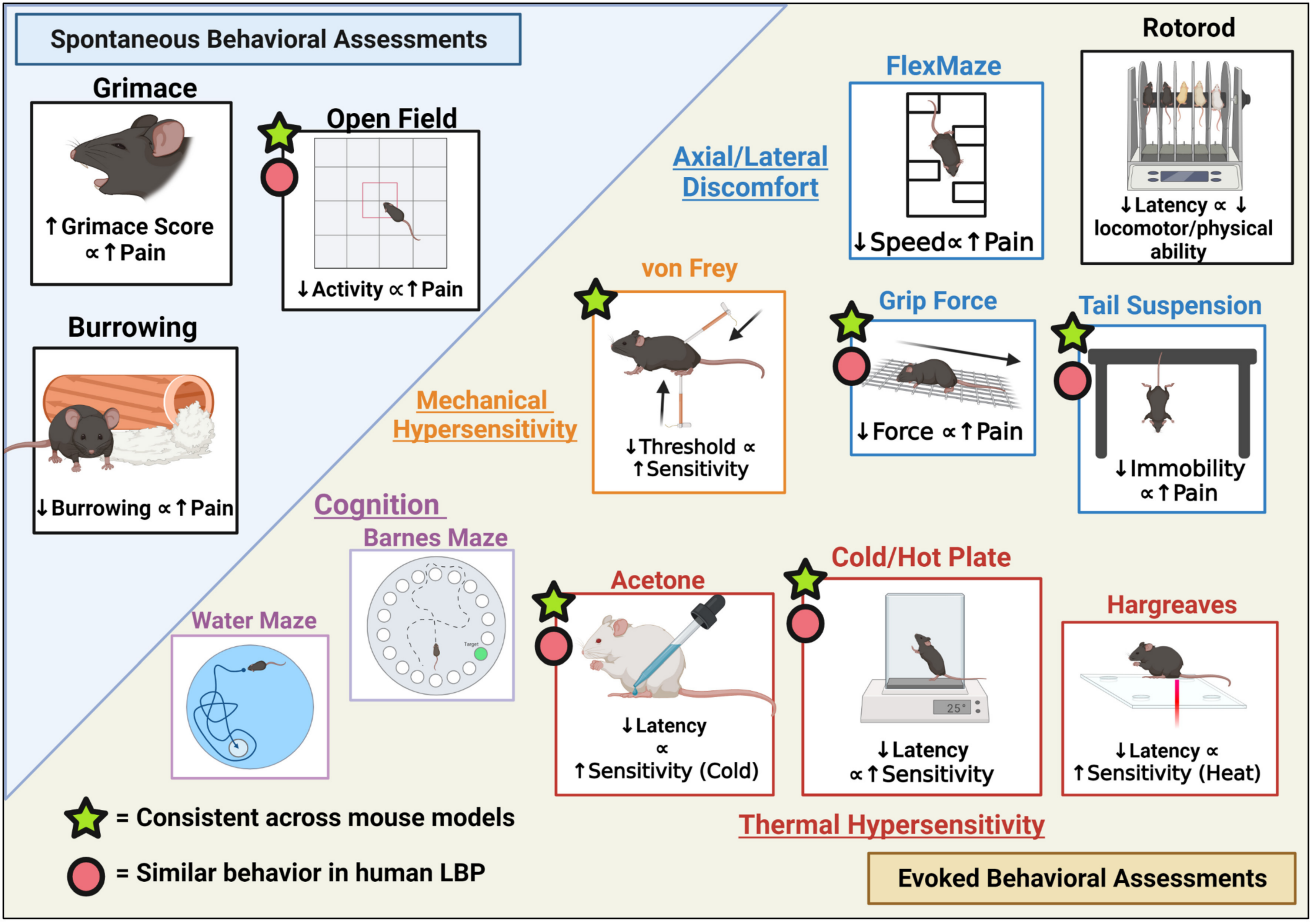
Pain-like Behavioral assessment with relevance to LBP categorized as Spontaneous vs. Evoked behavioral assessments and respective measures. Figure created using BioRender.com.

Pain-like behavioral assessments can be classified into evoked or spontaneous pain as illustrated in Figure 2. Spontaneous pain occurs in the absence of specific stimuli and is more indicative of clinical chronic pain conditions (170). Examples of spontaneous pain in mice may include audible vocalization, avoidance, and self-mutilation (171). Meanwhile, in evoked behavioral assessments, mice are presented with a stimulus representing sensory modalities that allow for the measurement of pain thresholds (172). Signs of evoked pain may include motor reflexes such as limb withdrawal from the stimulus, reduced locomotion, or agitation. Evoked pain can further be categorized into hyperalgesia and allodynia. According to the International Association for the Study of Pain (IASP), allodynia is “pain due to a stimulus that does not normally provoke pain” while hyperalgesia is “increased pain from a stimulus that normally provokes pain” (173). The following sections describe the different behavioral assessments used in rodent models of LBP and their associations with human pain.

#### Spontaneous Pain Behavior Assessments

Spontaneous behavioral assessments include open field, gait analyses, burrowing, and grimace scales. **Open field** involves the placement of mouse on a square field and allowing free-roaming to assess exploratory behavior with quantification of multiple parameters such as the number of times an area was visited, movement/sedentary time, and rearing (174). **Gait and weight-bearing analyses** are used to measure nociception in mice as ambulation can mechanically stimulate the spine and alter the way mice walk and bear weight. Several platforms are available for mice as reviewed by Deuis et al. (152). A limitation of weight-bearing analysis for the study of LBP is that these assessments may be easier to interpret for unilateral injury models comparing differences between uninjured vs. injured sides rather than an overall assessment of gait. **Grimace** (that includes orbital tightening, nose bulge or cheek bulk or ear position) can be observed and scored on a Grimace Scale as a measure of pain intensity in mice from a scale of 0 as being normal to 2 as in severe pain (severely altered facial features) (175). While the grimace test is considered accurate, a significant level of pain is required to detect these facial changes and is better utilized for pain of acute/moderate duration, not chronic pain such as LBP. In addition to grimace, **Paw behaviors** can be assessed but can be unreliable as the lifting behavior is not observed universally in pain models (16). **Burrowing** can be used to measure nociception where burrowing material is placed in the mouse cage and the burrowed material quantified before and after the mouse is introduced into the cage (176). Rodents experiencing pain or discomfort will burrow less compared to normal mice.

#### Evoked Pain Behavior Assessments

The **von Frey** assay is commonly used to evaluate mechanical allodynia *via* application of calibrated monofilaments or a handheld device to the plantar surface with evoked behaviors quantified (152). The **Tail Flick** test involves a heat stimulus to the mouse tail *via* a direct light beam or dipping the tail in hot water (~46–52°C) until a tail-flick is elicited (177). However, this also requires mouse restraint (may cause significant stress) and clinical translatability of this assessment is unclear (may be a spinal reflex rather than a pain response) (152). **A cold or hot plate** can be used to determine thermal hyperalgesia and involves placing mice on a heated or cooled plate and recording time for paw withdrawal, licking, stamping, leaning, or jumping in response to the stimulus. Alternatively, dynamic cold/hot plate tests can be used where the mouse is placed on the plate at non-noxious temperatures with the temperature increased/decreased and the temperature at which the mouse responds is recorded (152). The **Hargreaves** test uses radiant or an infrared heat stimulus aimed at the planter paw of the mouse through a glass platform and the time to withdraw is recorded (178). This test is preferred to the hot plate test as it allows the measurement of individual ipsilateral and contralateral heat thresholds while the cold or hotplate applies the stimulus to both paws at the same time (179). Similarly, if the goal is to record the temperature threshold rather than latency to static temperature, a **thermal probe** test can be used where the mouse is placed on a wire mesh where a probe of increasing temperature is applied to the paw and temperature at withdrawal is recorded (180). The **acetone** evaporation test is a measure of thermal allodynia and involves applying acetone to the mouse plantar paw surface with the number of evoked responses or severity of responses recorded (181). This is advantageous to the cold plate as a unilateral application can be achieved. Similarly, a **cold plantar** assay can be deployed *via* the application of dry or wet ice to the mice planter paw with the latency to withdrawal recorded for quantification of cold allodynia and hyperalgesia (182).

**Grip Force** is used to assess neuromuscular activity in rodents and is an indicator of muscle inflammation and deep tissue pain (183). The test uses grip meters, placing the mouse on a grip or rod, then the mouse is pulled back by the tail to induce stretching, and the peak force at the point of release is recorded. Similarly, an alternative is the **hanging wire** test where mice are placed on a wire mesh with grip induced before platform inversion and the latency to fall recorded (184). Decreased latency to fall would imply decreased motor function and increased pain. The **tail suspension** assay involves the suspension of mice by the tails (taped to the edge of a ridged platform) and the immobility/mobile time, rearing, full-extension, amongst other behaviors can be analyzed. While this test has been historically used as a measure of depression in mice, Millecamps et al. have used this as an assessment of axial pain (54, 185). More mobility will be observed in mice with axial stretching sensitivity due to pain compared to normal mice. **FlexMaze** assays were first utilized by Millecamps et al. where the mouse was placed in a maze and forced to undergo lateral flexion as they maneuvered their way through the maze (54). This allows for the measurement of lateral-flexion discomfort in mice similar to lateral human flexion. For locomotor capacity, a rotarod assessment may be used (186).

#### Cognitive Assessments

Although not utilized yet in mouse models of LBP, cognitive assessments such as the Barnes maze and Morris water maze may be useful to correlate DBP with psychosocial or nervous system injury/impairment (187). Although impairment in cognitive function due to LBP is rare, Schiltenwolf et al. revealed that patients with chronic LBP have slowed speeds of information processing and working memory (188).

### Comparison of Pain Assessments Between Models

Unlike *ex vivo* assessments of DRGs which have been widely utilized, behavioral assessments of pain in mice models of DBP are less well characterized. Only 35% of the papers included in this review assess pain as a major outcome and a majority of these use the SPARC-null mouse (54, 103, 104, 189). Thus, published models measuring pain-like behaviors are limited to the ventral lumbar puncture model, SPARC-null, and aged wild-type mice as shown in Table 3. Male or female mice were used ranging from 6 weeks to 2 years old with one study comparing both sexes (50) in terms of behavioral assessments. Results for spontaneous pain & locomotor capacity, mechanical & thermal sensitivities, and axial/lateral pain are described below.

#### Spontaneous Pain and Locomotor Capacity

Open field assessments of aged wild-type mice showed decreased rearing (time spent on hind limbs) with age and a decline in general activity similar to SPARC-null and injured mice with the addition of decreased burrowing in the puncture model after 4 weeks (50, 54, 84). This suggests that SPARC-null and injured mice experience spontaneous pain similar to age-related degeneration evidenced by decreased movement and rearing which requires significant trunk stability and lower body strength. Locomotor function was assessed in SPARC-null mice with interesting results between studies. In a study comparing 3 and 9-month-old SPARC-null to wild-type age-matched controls mice on the rotarod, no differences between the groups were observed which suggested no generalized nervous system dysfunction (105). In studies including male SPARC-null mice ranging from 6 to 78 weeks in age, rotarod assessment demonstrated improvement with age in SPARC-null mice and wild-type controls but drastically decreased after 70 weeks in SPARC-null mice while wild-type mice plateaued and the authors suggest this may be due to learned behavior on the rotarod (54). In female mice, within the same age range, rotarod activity suggested decreased physical function with age in SPARC-null and wild-type mice with no effect of strain (169).

#### Mechanical and Thermal Sensitivity

No differences in mechanical sensitivity were identified by von-Frey in SPARC-null mice compared to wild-type mice or effects of aging/time although some slight sensitivity was observed on the lower back (54, 103–105). Meanwhile, mice with multiple injured IVDs showed a significant decrease in threshold sensitivity at 12 weeks, suggesting increased mechanical sensitivity and mechanical allodynia while single level puncture CD-1 mice did not exhibit any difference (84, 85). Cold sensitivity assessed *via* acetone demonstrated increased evoked behaviors in female mice with punctured IVDs and in young male SPARC-null mice increasing hypersensitivity correlated with age. Interestingly, female SPARC-null mice exhibited cold hypersensitivity later at 18 weeks (54, 85, 104). In SPARC-null mice tail immersion was used to assess cold sensitivity but no differences were identified. Hot Plate, Hargreaves, Tail flick, and capsaicin were used as measures of heat sensitivity. Injured mice demonstrated decreased latency by 8 weeks post-op while SPARC-null mice exhibited no difference between young/old or wild-type mice in heat sensitivity, which highlights potential differences in pain mechanisms between injury puncture and genetic models. This is further supported by aged wild-type mice that demonstrated no correlation of thermal hyperalgesia to age, sex, or weight of mice (50).

#### Axial and Lateral Pain

Axial discomfort assessed by grip force or tail suspension in SPARC-null mice and mice with single IVD level injury showed decreased resistance to force along with decreased time immobile and increased time in self-supporting modes, suggesting SPARC-null and injury mice may experience axial discomfort and attempt to mitigate this through behaviors to alleviate axial stretch. This effect has also been observed in old wild-type mice (50). The FlexMaze demonstrated decreases in physical function associated with lateral flexion and reduced exploration speeds in SPARC-null mice (54). These assessments have not been made on mechanically induced or IVD puncture mice models.

### Translatability of Model Results to the Human Condition

A major difference between pain assessments in humans and animal is the inability of animals to communicate their pain. Humans can verbally communicate pain while for animals we rely on changes in pain-like behaviors as a proxy for pain. Unlike mouse models, humans have environmental influencers to pain and pain is also subjective as pain tolerances are different across patients. Physicians have clinical patient history and they provide in-depth physical examinations with pain scales (Numeric Pain Rating Scale (NPRS), Pain disability index (PDI), Visual Analog scale (VAS), and Oswestry index). Humans also possess psychosocial aspects which can affect pain while mice may not. However, some studies suggest mice have the potential for high-level emotional pain (190). As reviewed by Mogil et al., evoked assessments often fail in their translation to the clinic and thus spontaneous pain may be more clinically relevant to the human condition (191). It is evident from closer observation of mouse behavioral assessments, especially in evoked assessments, that the variability is heightened, and multiple factors can attribute to differences in pain-like behavior such as testing environment, the force of pull such as in grip tests, experimenter, and animal stress. Such factors need to be considered when assessing pain-like behaviors.

Neuropeptides such as CGRP have been identified in small DRG neurons involved in pain perception (192), and NPY is also upregulated in nerve injury/inflammation and present in the lumbar IVDs in humans and mice (193, 194). In addition, sodium ion channels are phosphorylated post-injury which allows for increased nociceptive signaling due to greater current density (195, 196). In direct molecular comparison of mice and human DRGs, TRK receptors present in small nociceptive neurons and TRPV1 were similar between humans and mice, while human neurons have a larger average size (~1.5–3 times larger than the mouse) (197). The mice models above demonstrated increased expression of neuropeptides along with increased sodium channels as assessed by IHC and RT-qPCR. Interestingly, receptor tyrosine kinase (RET), a pivotal protein in neuronal development, and Nav1.8/1.9 are significantly more abundant in TRKA+ cells of human DRGS compared to mice (197). Localization of CGRP and TRPV1 differs between humans and rodents (198) as does Nav1.8 activity (198, 199). This suggests that, while changes in specific markers may translate to human pain, their localization with in neuronal populations between species may be distinct.

Synonymous with open field and rotarod assessments, human patients with radiculopathy have slower gait speed, shorter travel distances, and greater standing time similar to aged old mice (44–47, 200). Patients with LBP have increased movement evoked fatigue, decreased physical activity, as well as reduced flexibility (201). In humans, mechanical hyperalgesia may also be measured similar to algometers in mice with the application of force with gradual intensity to the patient's lower back (43). Interestingly, mechanical allodynia is more relevant in patients with radicular pain due to nerve compression or inflammation which may explain the lack of differences when conducting von-Frey on mice models with IVD injury or genetic disposition that lack direct neuronal trauma (202). In terms of thermal hyperalgesia, humans with LBP may experience coldness, radiating pain, and cold allodynia in one or both legs which correlates with findings in mice using the cold plate and acetone tests along with the mouse models of IVD degeneration in this review (24, 203). Grip force and tail suspension tests showed axial discomfort which is also relevant in humans and sensitivity to stretching in mice models may be predictive of lumbar stiffness as perceived in humans (204). Additionally, mice models of IVD degeneration under tail suspension tend to have decreased time immobile with more time spent mitigating pain which is similar to the human condition where humans are more likely to modify movements to avoid pain than patients without pain (200, 205, 206). It is noteworthy to mention that “pain” is complex and multifaceted and cannot be directly measured in animal models. However, surrogate indirect measures such as observations of pain-like behaviors can be used to provide insight into DBP and the potential translation of therapies for LBP. Overall, the direct translation of pain in mice to human pain requires further interrogation, and advances such as multi-institutional collaborations involving multiple disciplines are critical before we can fully interpret the translation between models (207).

### Critical Pain Assessments for Mouse Models of IVD Degeneration

Based on the review of pain-like behaviors described above, we have identified key *ex vivo* and *in vivo* pain-like behaviors that can be utilized in mouse models. Given their similarity to humans and consistency across models, *ex vivo* assessment of CGRP, TRKA, TRPV1, and sodium channels levels are recommended. For *in vivo* assessments, the recommendations are derived from the small number of studies that assessed pain-like behaviors due to their consistent results and an increase in the number of studies may further elucidate more critical behavioral parameters. *In vivo* assessments could include open field for spontaneous pain-like behaviors, grip force & tail suspension for axial discomfort, and cold plate/acetone due to their similarity to human pain outcomes and consistency across models. Thermal hypersensitivity measures are less common in humans and have demonstrated inconsistencies across studies. While mechanical sensitivity is relevant in humans, the lack of significant difference in von-Frey in mice limits its clinical potential.

## Discussion/Conclusion and Future Perspectives

### Criteria for Choosing the Optimal Model

In this review, we have highlighted several mouse models of IVD degeneration with the potential for understanding disease mechanisms and for screening regenerative therapies for LBP *in vivo*. Methods of assessment on the molecular, cellular, tissue, structure/function, and pain level were discussed and highlighted in light of their clinical translation to LBP in humans. Several prominent induction methods and downstream parameters stood out between the assessed mouse models. Mechanically induced models such as those which use compression, instability, whole-body vibrations, and bipedalism along with needle puncture models demonstrated IVD degeneration at early time points compared to naturally aging mice and may be more clinically relevant as an acute model of IVD degeneration following herniation/trauma with controlled level-specific effects. These models also indicate that the mode of puncture is critical with changes observed with different sized needle gauges which alters the degree of degeneration (79). Genetic/aging models such as ERCC1, SM/J, and SPARC-null mice exhibited hallmarks of IVD degeneration at early ages compared to aged wild-type mice without forced trauma on the IVD and may be good models for spontaneously occurring chronic LBP. Clinical limitations to note include the fact that cause of accelerated aging in these mice may be due to developmental/global defects. IVD degeneration was present across all lumbar IVDs in genetic models (varying in degree of degeneration) such as the global SPARC-null mice while in humans, IVD degeneration related to LBP is more prevalent in lower lumbar levels (L4/L5, L5/S1) (54).

In consensus, model selection criteria are largely dependent on the research question and regenerative therapy assessed. For example, critical differences between mice and human IVDs are their small size and reduced diffusion of nutrients across the CEP. Therefore, they may not be optimal for studying cell-based therapies as the cell viability in a mouse model will likely differ in larger human IVDs. Rather, mice may serve as a good tool for studying small molecule therapies, drug evaluation, and non-viral gene delivery (83, 101). For example, the SPARC-null mice has been used for studying potential therapies targeting the IL-8 pathway and toll-like receptor 4 inhibition (189, 208). Another important limiting factor is the presence of notochordal cells in mouse models, however, accelerated aging models do demonstrate a shift of notochordal to NP chondrocyte-like cells such as in SM/J mice (56). Another research question relates to the stage/severity of degeneration the therapy is targeting. For example, mice models of IVD degeneration caused by mechanical manipulation or direct puncture typically develop degenerative changes relatively quickly (which can vary depending on the degree of mechanical forces/puncture, needle size or injection of pro-inflammatory cytokines) while genetic models may be more representative of slower developing IVD degeneration as a result of aging. Thus, the target population should be considered as in humans, elderly patients present with lumbar spinal stenosis due to a decrease in NP hydration and narrowed IVD height while younger patients are more prone to AF rupture and NP herniation (30, 209). Despite some limitations, these mice models are advantageous in their wide array of molecular assessments and genetic phenotypes and may serve as an excellent intermediate model between *in vitro* and more clinically relevant but logistically challenging large models such as the chondrodystrophic dog (49). They provide the ability to assess IVD structure/function and pain parameters in living animals whilst providing a more efficient way to screen regenerative therapies without using large animals, further contributing to the 3R principles (Reduce, Replacement, Refinement).

### Recommended Downstream Parameters

**Figure 3:**
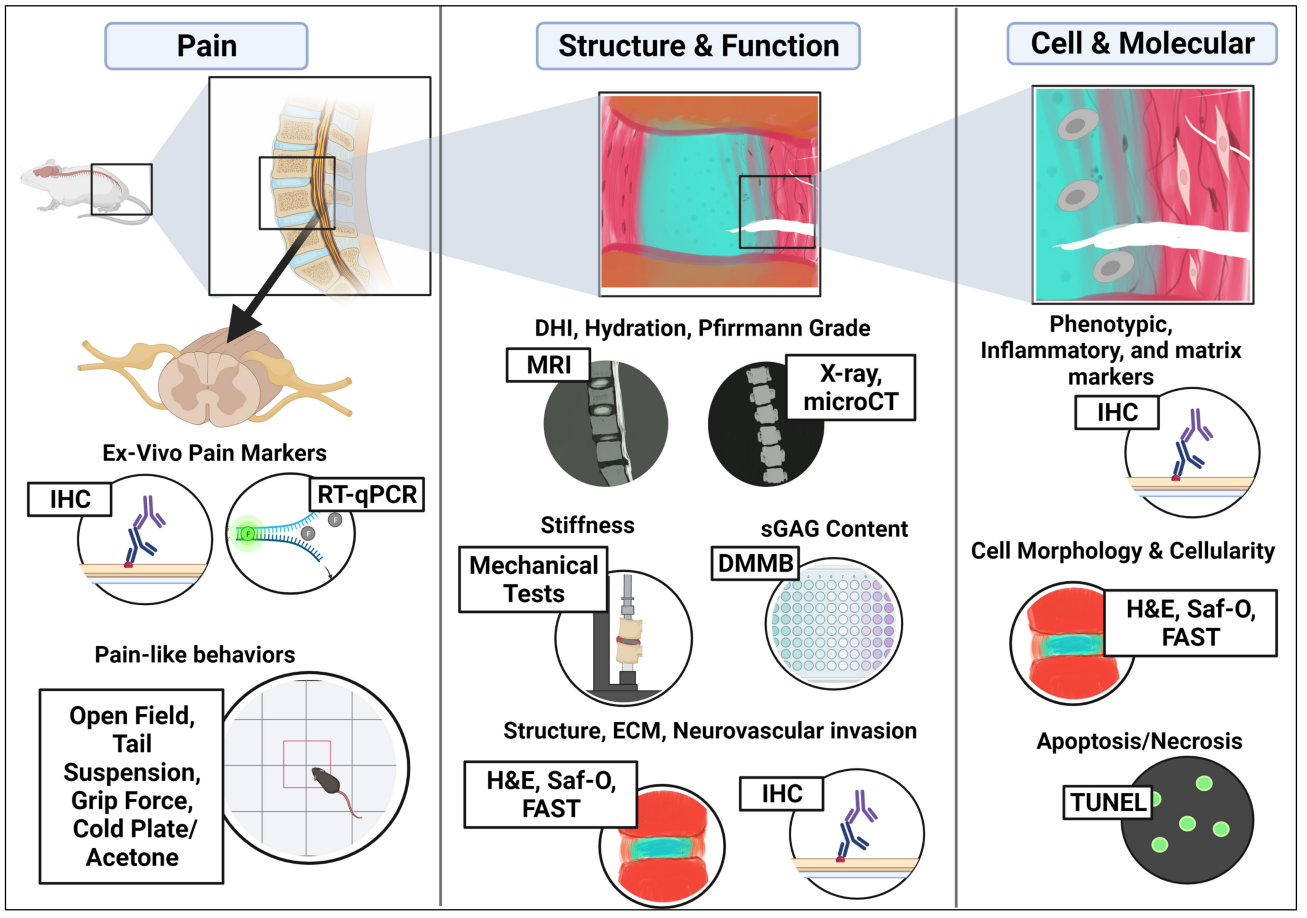
Critical assessments in mouse models of IVD degeneration for regenerative therapies used to treat Low Back Pain. Figure created using BioRender.com.

This review highlights critical cellular/molecular, tissue structure/functional, and pain assessments for determining the validity and efficacy of regenerative therapies in the mouse model as illustrated in Figure 3. Of significance, sex differences in pain perception and IVD degeneration have been found in humans as well as animal models inclusive of articles within this review, which warrants the need to include both male and females studies to accurately represent the clinical patient population (19, 210–212). It is also imperative as sex bias in animal testing has resulted in clinical limitations and only 10% of the studies presented here assessed sex differences as shown in Table 4, which highlights the percentage of models in this review assessing each parameter. Evidently, the parameters that were included most assessed IVD morphology (85%) and DHI & hydration (66%) which are critical parameters used to determine IVD structure and function. Spontaneous pain, neuronal function, mechanics, and locomotor capacity were parameters that were least assessed (<25%). While these measures are important, there under use in mouse models of LBP may be a result of a lack of investigator access to the proper tools/expertise. A solution maybe to enhance collaborations amongst research groups with differing areas of expertise so that these outcome measures can be included in more mouse models. In terms of timeline, IVD models saw effects as early as 1-week post-induction/injury or as early as 8 weeks in accelerated aging mice. However, results at earlier time points in mechanically induced or puncture models may be more related to acute inflammation and thus may not accurately represent IVD degeneration in the human. Longer time points are recommended with incremental time points if logistically possible (i.e., 4, 8, 12, weeks vs. end time point) to study the progression of the disease model over time and therapeutic outcomes both short and long term.

**Table 4:** Summary of assessed parameters across mouse models of IVD degeneration.

			**Molecular and cellular assessments**						**Tissue, structural, and functional assessments**						**Pain/behavioral assessments**							
**Model type**	**Reference**	**Sex inclusion**	**Phenotypic markers &**	**inflammatory cytokines**	**Cell morphology**	**Cellularity**	**Cell specific ecm markers**	**Matrix catabolism**	**Disc height index and hydration**	**Tissue level ecm content**	**Neurovascular invasion**	**Disc morphology**	**Degeneration grade**	**Mechanics**	**Neuronal assessment**	**Spontaneous pain**	**Locomotor**	**Mechanical sensitivity**	**Cold sensitivity**	**Heat sensitivity**	**Axial discomfort**	**Lateral flexion**
**Mechanically Induced**	(99)	M			**X**	**X**	**X**					**X**		**X**								
	(78)	M	**X**			**X**	**X**	**X**		**X**		**X**	**X**									
	(74)	F										**X**	**X**									
	(69)	M					**X**	**X**	**X**	**X**		**X**	**X**									
	(76)	M			**X**	**X**	**X**	**X**	**X**			**X**	**X**									
	(73)	M				**X**	**X**	**X**	**X**	**X**		**X**		**X**								
	(100)	N/R							**X**			**X**	**X**									
**Puncture**	(86)	N/R	**X**		**X**	**X**	**X**		**X**	**X**		**X**	**X**									
	(101)	N/R							**X**	**X**		**X**										
	(79)	N/R								**X**		**X**		**X**								
	(84)	F	**X**		**X**				**X**	**X**	**X**	**X**	**X**		**X**	**X**		**X**	**X**	**X**		
	(85)	F				**X**			**X**	**X**	**X**	**X**					**X**	**X**	**X**		**X**	
	(102)	N/R							**X**			**X**	**X**									
**Genetic/Aging**	(105)	M															**X**	**X**	**X**	**X**	**X**	
	(54)	M							**X**			**X**	**X**			**X**	**X**	**X**	**X**	**X**	**X**	**X**
	(55)	N/R				**X**	**X**	**X**	**X**	**X**												
	(103)	M									**X**				**X**		**X**	**X**	**X**	**X**	**X**	
	(104)	F										**X**	**X**				**X**	**X**	**X**		**X**	
	(56)	M/F	**X**		**X**	**X**	**X**	**X**	**X**	**X**		**X**	**X**	**X**								
	(50)	M/F							**X**		**X**	**X**			**X**	**X**			**X**	**X**	**X**	
**Percent of Studies Assessing Parameter** **→**		**10 %**	**20 %**		**25 %**	**40 %**	**40 %**	**30 %**	**66 %**	**50 %**	**20 %**	**85 %**	**55 %**	**20 %**	**15 %**	**15 %**	**25 %**	**30 %**	**35 %**	**25 %**	**30 %**	**5 %**

* *Key: M, male; F, female; N/R, sex of mice used in study not reported; X, assessment performed in respective article, percent of studies assessment parameter is calculated based on papers assessing parameters over the total number of papers included*.

The review of specific outcome measures provides a comprehensive overview of IVD degeneration in the mouse model as well as the potential to screen of regenerative therapies. As illustrated in Figure 3, these include **(****1****)** Histology and IHC to determine changes in disc structure and ECM, including nerve and immune cell infiltration, **(****2****)** Molecular and biochemical changes in ECM (proteoglycan and collagen) *via* DMMB or Hydroxyproline/Sircol assays, **(****3****)** Imaging IVD joints using microCT and MRI to assess changes in DHI, disc hydration, and grade, **(****4****)** Mechanical assessments for compressive stiffness, and **(****5****)** Pain-like behaviors such as open field, grip force, tail suspension, and cold plate/acetone tests. Histological assessments were used in most mice models to determine histopathological degeneration scores, structural integrity, disc height, and cellularity/cell morphology. IHC allows for the spatial assessment of specific proteins of interest and can be used to quantify changes in ECM matrix proteins, catabolic proteins, and even immune infiltration and neurovascular invasion into the IVD joint or neuronal structures such as innervating DRGs and spinal cord *ex vivo* as proxies of pain. Assays such as DMMB allow for quantitative assessments of proteoglycans in mice IVDs which is directly related to IVD hydration while TUNEL assays give information on changes in cellularity and cell death. Imaging methods such as microCT and MRI can be performed *in vivo* or *ex vivo* to quantify changes in IVD joint structure including DHI, DWI, hydration by disc intensity, as well as assessment of bone health adjacent to the IVDs. Lastly, as a major goal of regenerative therapies for IVD degeneration is to treat clinically relevant LBP, pain parameters *in vivo* are extremely critical in addition to *ex vivo* assessments.

In conclusion, this review has highlighted existing models of IVD degeneration for translational research and the treatment of LBP (acute vs. chronic), compared/contrasted induction methods (Mechanical, injury, genetic, age), and has outlined critical methods/parameters for both characterizing disease and downstream assessment of regenerative therapies in the mouse model. We hope this review will assist with model selection and critical parameters for assessments within the LBP research community to further push the translatability of clinically relevant therapies.

## Author Contributions

All authors contributed to this manuscript in the literature review, drafting and review of the article, intellectual contributions in their respective disciplines, and have approved the final version to be submitted.

## Funding

This review was supported by the Ohio State University (OSU) Department of Biomedical Engineering, The Department of Orthopedics, and the OSU Rodent Behavioral Core (P30NS045758).

## Conflict of Interest

The authors declare that the research was conducted in the absence of any commercial or financial relationships that could be construed as a potential conflict of interest.

## Publisher's Note

All claims expressed in this article are solely those of the authors and do not necessarily represent those of their affiliated organizations, or those of the publisher, the editors and the reviewers. Any product that may be evaluated in this article, or claim that may be made by its manufacturer, is not guaranteed or endorsed by the publisher.

## Abbreviations

AB: Alcian blue
ADAMTs: ADAM Metallopeptidase
AF: Annulus Fibrosus
BDL: Bile duct ligation
BDNF: brain-derived neurotrophic factor
CA#: Carbonic Anhydrase #
CEP: Cartilage Endplate
CD#: Clustoer of Differention #
CGRP: Calcitonin gene-related peptide
CTGF/CCN2: Connective Tissue Growth Factor/ Cellular Communication Network Factor 2
DBP: Discogenic Back Pain
DHI: Disc Height Index
DIPEN, DMMB: dimethymethyline blue assay
DNA: Deoxyribonucleic acid
DRG: Dorsal Root Ganglion
DWI: Disc width index
ECM: Extracellular Matrix
ELN: Elastin
ERCC1: Excision Repair 1
ERK: extracellular signal-regulated protein kinase
FAST: combination of fast green, Alcian blue, Safranin-O, and tartrazine stains
FMOD: Fibromodulin
FTIR: Fourier-transform infrared spectroscopy
FOX#: Forkhead Box #
GAG: Glycosaminoglycan
GDF5: Growth differentiation factor 5
GFAP: Glial fibrillary acidic protein
GFP: Green Fluorescent Protein
GLUT-1: Glucose transport 1
H&E: Hematoxylin & Eosin
IF: immunofluorescence
IHC: immunohistochemistry
IL-1β: Interleukin-1 Beta
IL-6: Interleukin 6
IVD: Intervertebral Disc
KRT: Keratin
LBP: Low back pain
microCT: micro-computed tomography
MKX: Mohawk
MMP: Matrix Metalloproteinase
MRI: magnetic resonance imaging
NGF: Nerve Growth Factor
NP: Nucleus Pulposus
NPRS: Numeric Pain Rating Scale
NPY: Neuropeptide Y
NSAIDs: Non-steroidal anti-inflammatory drugs
P16NK: p16 tumor suppressor gene
PAR2: protease-activated receptor 2
PDI: Pain disability index
PGP9.5: Protein Gene Product 9.5
PSR: Picrosirius red
RET: Receptor tyrosine Kinase
RNA: Ribonucleic acid
RUNX2: RUNX Family Transcription Factor 2
RT-qPCR: quantitative reverse transcription polymerase chain reaction
SCX: Scleraxis
SPARC-null: secreted protein acidic and rich in cysteine-null
SOX: SRY-Box Transcription Factor
SDC4: Syndecan 4
TIMP1: Tissue inhibitor matrix metalloproteinase 1
TNFα: Tumor necrosis factor alpha
TrKA: Tropomyosin receptor kinase A
TRPV1: transient receptor potential cation channel subfamily V member 1
TUNNEL: Terminal deoxynucleotidyl transferase dUTP nick end labeling
VAS: Visual analog scale
VEGF: Vascular endothelial growth factor
VGSCs: Voltage-gated sodium channels.

## References

[1] Global Burden of Disease Study 2013 Collaborators. Global, regional, and national incidence, prevalence, and years lived with disability for 301 acute and chronic diseases and injuries in 188 countries, 1990–2013: a systematic analysis for the Global Burden of Disease Study 2013. Lancet. (2015) 386:743–800. 10.1016/S0140-6736(15)60692-4 26063472 PMC4561509

[2] Freburger JK Holmes GM Agans RP Jackman AM Darter JD Wallace AS. The rising prevalence of chronic low back pain. Arch Intern Med. (2009) 169:251–8. 10.1001/archinternmed.2008.543 19204216 PMC4339077

[3] Katz JN. Lumbar disc disorders and low-back pain: socioeconomic factors and consequences. J Bone Jt Surg. (2006) 88:21–4. 10.2106/00004623-200604002-00005 16595438

[4] Chou R Qaseem A Snow V Casey D Cross TJ Shekelle P. Diagnosis and treatment of low back pain: a joint clinical practice guideline from the American College of Physicians and the American Pain Society. Ann Intern Med. (2007) 147:478–91. 10.7326/0003-4819-147-7-200710020-00006 17909209

[5] Lu Y Guzman JZ Purmessur D Iatridis JC Hecht AC Qureshi SA Cho SK. Nonoperative management of discogenic back pain. Spine. (2014) 39:1314–24. 10.1097/BRS.0000000000000401 24827515 PMC4144979

[6] Zhao L Manchikanti L Kaye AD Abd-Elsayed A. Treatment of discogenic low back pain: current treatment strategies and future options-a literature review. Curr Pain Headache Rep. (2019) 23:86. 10.1007/s11916-019-0821-x 31707499

[7] Deyo RA Von Korff M Duhrkoop D. Opioids for low back pain. BMJ. (2015) 350:g6380. 10.1136/bmj.g6380 25561513 PMC6882374

[8] Schwarzer AC Aprill CN Derby R Fortin J Kine G Bogduk N. The prevalence and clinical features of internal disc disruption in patients with chronic low back pain. Spine. (1995) 20:1878–83. 10.1097/00007632-199509000-00007 8560335

[9] Luoma K Riihimäki H Luukkonen R Raininko R Viikari-Juntura E Lamminen A. Low back pain in relation to lumbar disc degeneration. Spine. (2000) 25:487–92. 10.1097/00007632-200002150-00016 10707396

[10] Wang HQ Samartzis D. Clarifying the nomenclature of intervertebral disc degeneration and displacement: from bench to bedside. Int J Clin Exp Pathol. (2014) 7:1293–8. 24817926 PMC4014210

[11] Buckwalter JA. Spine update: aging and degeneration of the human intervertebral disc. Spine. (1995) 20:1307–14. 10.1097/00007632-199506000-00022 7660243

[12] Jin L Balian G Li XJ. Animal models for disc degeneration-an update. Histol Histopathol. (2018) 33:543–54. 10.14670/HH-11-910 28580566 PMC5975243

[13] Alini M Eisenstein SM Ito K Little C Kettler AA Masuda K. Are animal models useful for studying human disc disorders/degeneration? Eur Spine J. (2008) 17:2–19. 10.1007/s00586-007-0414-y 17632738 PMC2365516

[14] Lotz JC. Animal models of intervertebral disc degeneration. Spine. (2004) 29:2742–50. 10.1097/01.brs.0000146498.04628.f9 15564923

[15] Singh K Masuda K An HS. Animal models for human disc degeneration. Spine J. (2005) 5:S267–79. 10.1016/j.spinee.2005.02.016 16291123

[16] Mosley GE Evashwick-Rogler TW Lai A Iatridis JC. Looking beyond the intervertebral disc: The need for behavioral assays in models of discogenic pain. Ann N Y Acad Sci. (2017) 1409:51–66. 10.1111/nyas.13429 28797134 PMC5730458

[17] Lotz JC Ulrich JA. Innervation, Inflammation, and Hypermobility May Characterize Pathologic Disc Degeneration: Review of Animal Model Data. J Bone Jt Surg. (2006) 88:76–82. 10.2106/00004623-200604002-00016 16595449

[18] de Schepper EIT Damen J van Meurs JBJ Ginai AZ Popham M Hofman A. The association between lumbar disc degeneration and low back pain. Spine. (2010) 35:531–536. 10.1097/BRS.0b013e3181aa5b33 20147869

[19] Mosley GE Wang M Nasser P Lai A Charen DA Zhang B. Males and females exhibit distinct relationships between intervertebral disc degeneration and pain in a rat model. Sci Rep. (2020) 10:15120. 10.1038/s41598-020-72081-9 32934258 PMC7492468

[20] Sorge RE Totsch SK. Sex Differences in Pain. J Neurosci Res. (2017) 95:1271–81. 10.1002/jnr.23841 27452349

[21] Ghosh P. The Biology of the Intervertebral disc, volume 2. 1st ed. Boca Raton, FL: CRC Press (1988). p. 39–108.

[22] Trout JJ Buckwalter JA Moore KC Landas SK. Ultrastructure of the human intervertebral disc. I Changes in notochordal cells with age. Tissue Cell. (1982) 14:359–69. 10.1016/0040-8166(82)90033-7 7202266

[23] Trout JJ Buckwalter JA Moore KC. Ultrastructure of the human intervertebral disc: II. Cells of the nucleus pulposus. Anat Rec. (1982) 204:307–14. 10.1002/ar.1092040403 7181135

[24] Raj PP. Intervertebral disc: anatomy-physiology-pathophysiology-treatment. Pain Pract. (2008) 8:18–44. 10.1111/j.1533-2500.2007.00171.x 18211591

[25] Liang T Zhang L-L Xia W Yang H-L Luo Z-P. Individual collagen fibril thickening and stiffening of annulus fibrosus in degenerative intervertebral disc. Spine. (2017) 42:E1104–11. 10.1097/BRS.0000000000002085 28146016

[26] Holm S Maroudas A Urban JPG Selstam G Nachemson A. Nutrition of the intervertebral disc: solute transport and metabolism. Connect Tissue Res. (1981) 8:101–19. 10.3109/03008208109152130 6453689

[27] Freemont AJ. The cellular pathobiology of the degenerate intervertebral disc and discogenic back pain. Rheumatology. (2008) 48:5–10. 10.1093/rheumatology/ken396 18854342

[28] Le Maitre C Hoyland J Freemont AJ. Catabolic cytokine expression in degenerate and herniated human intervertebral discs: IL-1β and TNFα expression profile. Arthritis Res Ther. (2007) 9:R77. 10.1186/ar2275 17688691 PMC2206382

[29] Purmessur D Freemont AJ Hoyland JA. Expression and regulation of neurotrophins in the nondegenerate and degenerate human intervertebral disc. Arthritis Res Ther. (2008) 10:1–9. 10.1186/ar2487 18727839 PMC2575613

[30] Smith LJ Nerurkar NL Choi K-SK-SS Harfe BD Elliott DM. Degeneration and regeneration of the intervertebral disc: lessons from development. Dis Model Mech. (2011) 4:31–41. 10.1242/dmm.006403 21123625 PMC3008962

[31] Maroudas A Stockwell RA Nachemson A Urban J. Factors involved in the nutrition of the human lumbar intervertebral disc: cellularity and diffusion of glucose in vitro. J Anat. (1975) 120:113–30. 1184452 PMC1231728

[32] Gorth DJ Shapiro IM Risbud M V. Transgenic mice overexpressing human TNF-α experience early onset spontaneous intervertebral disc herniation in the absence of overt degeneration. Cell Death Dis. (2019) 10:7. 10.1038/s41419-018-1246-x 30584238 PMC6315044

[33] Jin L Xiao L Ding M Pan A Balian G Sung SSJ Li XJ. Heterogeneous macrophages contribute to the pathology of disc herniation induced radiculopathy. Spine J. (2022) 22:677–89. 10.1016/j.spinee.2021.10.014 34718176 PMC8957503

[34] Kawakubo A Uchida K Miyagi M Nakawaki M Satoh M Sekiguchi H. Investigation of resident and recruited macrophages following disc injury in mice. J Orthop Res. (2020) 38:1703–9. 10.1002/jor.24590 31965590

[35] Lee S Millecamps M Foster DZ Stone LS. Long-term histological analysis of innervation and macrophage infiltration in a mouse model of intervertebral disc injury–induced low back pain. J Orthop Res. (2020) 38:1238–47. 10.1002/jor.24560 31814143

[36] Richards J Tang S Gunsch G Sul P Wiet M Flanigan DC. Mast Cell/Proteinase Activated Receptor 2 (PAR2) Mediated Interactions in the Pathogenesis of Discogenic Back Pain. Front Cell Neurosci. (2019) 13:294. 10.3389/fncel.2019.00294 31333416 PMC6625229

[37] Wiet MG Piscioneri A Khan SN Ballinger MN Hoyland JA Purmessur D. Mast Cell-Intervertebral disc cell interactions regulate inflammation, catabolism and angiogenesis in Discogenic Back Pain. Sci Rep. (2017) 7:12492. 10.1038/s41598-017-12666-z 28970490 PMC5624870

[38] O'Connell GD Vresilovic EJ Elliott DM. Comparison of animals used in disc research to human lumbar disc geometry. Spine. (2007) 32:328–33. 10.1097/01.brs.0000253961.40910.c1 17268264

[39] Hunter CJ Matyas JR Duncan NA Ph D Matyas JR Ph D. The notochordal cell in the nucleus pulposus : a review in the context of tissue engineering. Tissue Eng. (2003) 9:667–77. 10.1089/107632703768247368 13678445

[40] Daly C Ghosh P Jenkin G Oehme D Goldschlager T. A review of animal models of intervertebral disc degeneration: pathophysiology, regeneration, and translation to the clinic. Biomed Res Int. (2016) 2016:1–14. 10.1155/2016/5952165 27314030 PMC4893450

[41] Pfirrmann CWA Metzdorf A Elfering A Hodler J Boos N. Effect of aging and degeneration on disc volume and shape: A quantitative study in asymptomatic volunteers. J Orthop Res. (2006) 24:1086–94. 10.1002/jor.20113 16609964

[42] Peloquin JM Yoder JH Jacobs NT Moon SM Wright AC Vresilovic EJ. Human L3L4 intervertebral disc mean 3D shape, modes of variation, and their relationship to degeneration. J Biomech. (2014) 47:2452–9. 10.1016/j.jbiomech.2014.04.014 24792581 PMC4115453

[43] Breivik H Collett B Ventafridda V Cohen R Gallacher D. Survey of chronic pain in Europe: prevalence, impact on daily life, and treatment. Eur J Pain. (2006) 10:287–287. 10.1016/j.ejpain.2005.06.009 16095934

[44] Fahlström A Yu Q Ulfhake B. Behavioral changes in aging female C57BL/6 mice. Neurobiol Aging. (2011) 32:1868–80. 10.1016/j.neurobiolaging.2009.11.003 20005598

[45] Houtkooper RH Argmann C Houten SM Cantó C Jeninga EH Andreux PA. The metabolic footprint of aging in mice. Sci Rep. (2011) 1:134. 10.1038/srep00134 22355651 PMC3216615

[46] Justice JN Carter CS Beck HJ Gioscia-Ryan RA McQueen M Enoka RM. Battery of behavioral tests in mice that models age-associated changes in human motor function. Age. (2014) 36:583–95. 10.1007/s11357-013-9589-9 24122289 PMC4039275

[47] Bair WN Petr M Alfaras I Mitchell SJ Bernier M Ferrucci L. Of Aging Mice and Men: Gait Speed Decline Is a Translatable Trait, with Species-Specific Underlying Properties. J Gerontol A Biol Sci Med Sci. (2019) 74:1413–6. 10.1093/gerona/glz015 30649206 PMC6696716

[48] Shiri R Falah-Hassani K Heliövaara M Solovieva S Amiri S Lallukka T. Risk factors for low back pain: a population-based longitudinal study. Arthritis Care Res. (2019) 71:290–9. 10.1002/acr.23710 30044543

[49] Thompson K Moore S Tang S Wiet M Purmessur D. The chondrodystrophic dog: a clinically relevant intermediate-sized animal model for the study of intervertebral disc-associated spinal pain. JOR Spine. (2018) 1:1–13. 10.1002/jsp2.1011 29984354 PMC6018624

[50] Vincent K Mohanty S Pinelli R Bonavita R Pricop P Albert TJ. Aging of mouse intervertebral disc and association with back pain. Bone. (2019) 123:246–59. 10.1016/j.bone.2019.03.037 30936040 PMC6549718

[51] Alvarez-Garcia O Matsuzaki T Olmer M Masuda K Lotz MK. Age-related reduction in the expression of FOXO transcription factors and correlations with intervertebral disc degeneration. J Orthop Res. (2017) 35:2682–91. 10.1002/jor.23583 28430387 PMC5650945

[52] Mason RM Palfrey AJ. Intervertebral disc degeneration in adult mice with hereditary kyphoscoliosis. J Orthop Res. (1984) 2:333–8. 10.1002/jor.1100020405 6527158

[53] Li X Leo BM Beck G Balian G Anderson DG. Collagen and proteoglycan abnormalities in the GDF-5-deficient mice and molecular changes when treating disk cells with recombinant growth factor. Spine. (2004) 29:2229–34. 10.1097/01.brs.0000142427.82605.fb 15480133

[54] M Millecamps MTLNESLS Millecamps M Tajerian M Naso L Sage EH Stone LS. Lumbar intervertebral disc degeneration associated with axial and radiating low back pain in ageing SPARC-null mice. Pain. (2012) 153:1167–79. 10.1016/j.pain.2012.01.027 22414871

[55] Vo N Seo HY Robinson A Sowa G Bentley D Taylor L. Accelerated aging of intervertebral discs in a mouse model of progeria. J Orthop Res. (2010) 28:1600–7. 10.1002/jor.21153 20973062 PMC3477798

[56] Choi H Tessier S Silagi ES Kyada R Yousefi F Pleshko N. A novel mouse model of intervertebral disc degeneration shows altered cell fate and matrix homeostasis. Matrix Biol. (2018) 70:102–22. 10.1016/j.matbio.2018.03.019 29605718 PMC6081256

[57] Kimura T Nakata K Tsumaki N Miyamoto S Matsui Y Ebara S. Progressive degeneration of articular cartilage and intervertebral discs: an experimental study in transgenic mice bearing a type IX collagen mutation. Int Orthop. (1996) 20:177–81. 10.1007/s002640050058 8832322

[58] Sahlman J Inkinen R Hirvonen T Lammi MJ Lammi PE Nieminen J. Premature vertebral endplate ossification and mild disc degeneration in mice after inactivation of one allele belonging to the Col2a1 gene for type II collagen. Spine. (2001) 26:2558–65. 10.1097/00007632-200112010-00008 11725236

[59] Boyd LM Richardson WJ Allen KD Flahiff C Jing L Li Y. Early-onset degeneration of the intervertebral disc and vertebral end plate in mice deficient in type IX collagen. (2008) 58:164–71. 10.1002/art.23231 18163498

[60] Furukawa T Ito K Nuka S Hashimoto J Takei H Takahara M. Absence of biglycan accelerates the degenerative process in mouse intervertebral disc. Spine. (2009) 34:E911–7. 10.1097/BRS.0b013e3181b7c7ec 19940720 PMC3125573

[61] Aszódi A Chan D Hunziker E Bateman JF Fässler R. Collagen II is essential for the removal of the notochord and the formation of intervertebral discs. J Cell Biol. (1998) 143:1399–412. 10.1083/jcb.143.5.1399 9832566 PMC2133086

[62] Watanabe H Nakata K Kimata K Nakanishi I Yamada Y. Dwarfism and age-associated spinal degeneration of heterozygote cmd mice defective in aggrecan. Proc Natl Acad Sci U S A. (1997) 94:6943–7. 10.1073/pnas.94.13.6943 9192671 PMC21264

[63] Battié MC Videman T. Lumbar disc degeneration: epidemiology and genetics. J Bone Jt Surg. (2006) 3–9 10.2106/00004623-200604002-00002 16595435

[64] Battié MC Videman T Levalahti E Gill K Kaprio J. Heritability of low back pain and the role of disc degeneration. Pain. (2007) 131:272–80. 10.1016/j.pain.2007.01.010 17335977

[65] Battié MC Videman T Kaprio J Gibbons LE Gill K Manninen H. The twin spine study: contributions to a changing view of disc degeneration†. Spine J. (2009) 9:47–59. 10.1016/j.spinee.2008.11.011 19111259

[66] Teraguchi M Yoshimura N Hashizume H Muraki S Yamada H Minamide A. Prevalence and distribution of intervertebral disc degeneration over the entire spine in a population-based cohort: the Wakayama Spine Study. Osteoarthr Cartil. (2014) 22:104–10. 10.1016/j.joca.2013.10.019 24239943

[67] Miyamoto S Yonenobu K Ono K. Experimental cervical spondylosis in the mouse. Spine. (1991) 16:S495–500. 10.1097/00007632-199110001-00008 1801260

[68] Court C Colliou OK Chin JR Liebenberg E Bradford DS Lotz JC. The effect of static in vivo bending on the murine intervertebral disc. Spine J. (2001) 1:239–45. 10.1016/S1529-9430(01)00056-0 14588327

[69] Oichi T Taniguchi Y Soma K Chang SH Yano F Tanaka S. A mouse intervertebral disc degeneration model by surgically induced instability. Spine. (2018) 43:E557–64. 10.1097/BRS.0000000000002427 29016437

[70] Walsh AJL Lotz JC. Biological response of the intervertebral disc to dynamic loading. J Biomech. (2004) 37:329–37. 10.1016/S0021-9290(03)00290-2 14757452

[71] Vergroesen PPA Emanuel KS Peeters M Kingma I Smit TH. Are axial intervertebral disc biomechanics determined by osmosis? J Biomech. (2018) 70:4–9. 10.1016/j.jbiomech.2017.04.027 28579261

[72] Lao Y Xu T Jin H Ruan H Wang J Zhou L. Accumulated spinal axial biomechanical loading induces degeneration in intervertebral disc of mice lumbar spine. Orthop Surg. (2018) 10:56–63. 10.1111/os.12365 29436145 PMC6594506

[73] Liu Z Zhou Q Zheng J Li C Zhang W Zhang X. A novel *in vivo* mouse intervertebral disc degeneration model induced by compressive suture. Exp Cell Res. (2021) 398:112359. 10.1016/j.yexcr.2020.112359 33221315

[74] Sakai D Nishimura K Tanaka M Nakajima D Grad S Alini M. Migration of bone marrow-derived cells for endogenous repair in a new tail-looping disc degeneration model in the mouse: a pilot study. Spine J. (2015) 15:1356–65. 10.1016/j.spinee.2013.07.491 25459743

[75] Higuchi M Abe K Kaneda K. Changes in the nucleus pulposus of the intervertebral disc in bipedal mice. A light and electron microscopic study. Clin Orthop Relat Res. (1983) 251–7. 6839597

[76] Ao X Wang L Shao Y Chen X Zhang J Chu J. Development and characterization of a novel bipedal standing mouse model of intervertebral disc and facet joint degeneration. Clin Orthop Relat Res. (2019) 477:1492–504. 10.1097/CORR.0000000000000712 31094848 PMC6554109

[77] Bailey JF Hargens AR Cheng KK Lotz JC. Effect of microgravity on the biomechanical properties of lumbar and caudal intervertebral discs in mice. J Biomech. (2014) 47:2983–8. 10.1016/j.jbiomech.2014.07.005 25085756

[78] McCann MR Patel P Pest MA Ratneswaran A Lalli G Beaucage KL. Repeated exposure to high-frequency low-amplitude vibration induces degeneration of murine intervertebral discs and knee joints. Arthritis Rheumatol. (2015) 67:2164–75. 10.1002/art.39154 25891852

[79] Martin JT Gorth DJ Beattie EE Harfe BD Smith LJ Elliott DM. Needle puncture injury causes acute and long-term mechanical deficiency in a mouse model of intervertebral disc degeneration. J Orthop Res. (2013) 31:1276–82. 10.1002/jor.22355 23553925 PMC6684036

[80] Michalek AJ Iatridis JC. Penetrating annulus fibrosus injuries affect dynamic compressive behaviors of the intervertebral disc via altered fluid flow: an analytical interpretation. J Biomech Eng. (2011) 133:84502. 10.1115/1.4004915 21950904 PMC3215273

[81] Tian Z Ma X Yasen M Mauck RL Qin L Shofer FS. Intervertebral disc degeneration in a percutaneous mouse tail injury model. Am J Phys Med Rehabil. (2018) 97:170–7. 10.1097/PHM.0000000000000818 28863006 PMC5823709

[82] Ohnishi T Sudo H Iwasaki K Tsujimoto T Ito YM Iwasaki N. *In vivo* mouse intervertebral disc degeneration model based on a new histological classification. PLoS ONE. (2016) 11:e0160486. 10.1371/journal.pone.0160486 27482708 PMC4970753

[83] Tang S Salazar-Puerta A Richards J Khan S Hoyland J Gallego-Perez D. Non-viral reprogramming of human nucleus pulposus cells with FOXF1 via extracellular vesicle delivery: an *in vitro* and *in vivo* study. Eur Cells Mater. (2021) 41:90–107. 10.22203/eCM.v041a07 33465243 PMC8514169

[84] Shi C Das V Li X Kc R Qiu S O-Sullivan I. Development of an in vivo mouse model of discogenic low back pain. J Cell Physiol. (2018) 233:6589–602. 10.1002/jcp.26280 29150945

[85] Millecamps M Stone LS. Delayed onset of persistent discogenic axial and radiating pain after a single-level lumbar intervertebral disc injury in mice. Pain. (2018) 159:1843–55. 10.1097/j.pain.0000000000001284 29794612

[86] Yang F Leung VY Luk KD Chan D Cheung KM. Injury-induced sequential transformation of notochordal nucleus pulposus to chondrogenic and fibrocartilaginous phenotype in the mouse. J Pathol. (2009) 218:113–21. 10.1002/path.2519 19288580

[87] Elliott DM Yerramalli CS Beckstein JC Boxberger JI Johannessen W Vresilovic EJ. The effect of relative needle diameter in puncture and sham injection animal models of degeneration. Spine. (2008) 33:588–96. 10.1097/BRS.0b013e318166e0a2 18344851

[88] Masuda K Aota Y Muehleman C Imai Y Okuma M Thonar EEJE. A novel rabbit model of mild, reproducible disc degeneration by an anulus needle puncture: correlation between the degree of disc injury and radiological and histological appearances of disc degeneration. Spine. (2005) 30:5–14. 10.1097/01.brs.0000148152.04401.20 15626974

[89] Michalek AJ Funabashi KL Iatridis JC. Needle puncture injury of the rat intervertebral disc affects torsional and compressive biomechanics differently. Eur Spine J. (2010) 19:2110–6. 10.1007/s00586-010-1473-z 20544231 PMC2997207

[90] Hsieh AHAAH Hwang D Ryan DA Freeman AKA Kim H. Degenerative anular changes induced by puncture are associated with insufficiency of disc biomechanical function. Spine. (2009) 34:998–1005. 10.1097/BRS.0b013e31819c09c4 19404174

[91] Sobajima S Kompel JF Kim JS Wallach CJ Robertson DD Vogt MT. A slowly progressive and reproducible animal model of intervertebral disc degeneration characterized by MRI, X-ray, and histology. Spine. (2005) 30:15–24. 10.1097/01.brs.0000148048.15348.9b 15626975

[92] Krishnamoorthy D Hoy RC Natelson DM Torre OM Laudier DM Iatridis JC. Dietary advanced glycation end-product consumption leads to mechanical stiffening of murine intervertebral discs. Dis Model Mech. (2018) 11:dmm036012. 10.1101/342691 30498097 PMC6307905

[93] Wang D Nasto LA Roughley P Leme AS Houghton AM Usas A. Spine degeneration in a murine model of chronic human tobacco smokers. Osteoarthr Cartil. (2012) 20:896–905. 10.1016/j.joca.2012.04.010 22531458 PMC3389285

[94] Liuke M Solovieva S Lamminen A Luoma K Leino-Arjas P Luukkonen R. Disc degeneration of the lumbar spine in relation to overweight. Int J Obes. (2005) 29:903–8. 10.1038/sj.ijo.0802974 15917859

[95] Takatalo J Karppinen J Taimela S Niinimäki J Laitinen J Sequeiros RB. Association of abdominal obesity with lumbar disc degeneration-a magnetic resonance imaging study. PLoS ONE. (2013) 8:e56244. 10.1371/journal.pone.0056244 23418543 PMC3571955

[96] Jakoi AM Pannu G D'Oro A Buser Z Pham MH Patel NN. The clinical correlations between diabetes, cigarette smoking and obesity on intervertebral degenerative disc disease of the lumbar spine. Asian Spine J. (2017) 11:337–47. 10.4184/asj.2017.11.3.337 28670401 PMC5481588

[97] Samartzis D Karppinen J Cheung JPY Lotz J. Disk degeneration and low back pain: are they fat-related conditions? Glob spine J. (2013) 3:133–44. 10.1055/s-0033-1350054 24436864 PMC3854598

[98] Fabiane SM Ward KJ Iatridis JC Williams FMK. Does type 2 diabetes mellitus promote intervertebral disc degeneration? Eur Spine J. (2016) 25:2716–20. 10.1007/s00586-016-4612-3 27272275 PMC5026921

[99] Lotz JC Colliou OK Chin JR Duncan NA Liebenberg E. Compression-induced degeneration of the intervertebral disc: An in vivo mouse model and finite-element study. Spine. (1998) 23:2493–506. 10.1097/00007632-199812010-00004 9854748

[100] Liu S Sun Y Dong J Bian Q. A mouse model of lumbar spine instability. J Vis Exp. (2021) 2021:e61722. 10.3791/61722 33970135

[101] Liang H Ma S-Y Feng G Shen FH Joshua Li X. Therapeutic effects of adenovirus-mediated growth and differentiation factor-5 in a mice disc degeneration model induced by annulus needle puncture. Spine J. (2010) 10:32–41. 10.1016/j.spinee.2009.10.006 19926342 PMC2818300

[102] Baldia M Mani S Walter N Kumar S Srivastava A Prabhu K. Development of a unique mouse intervertebral disc degeneration model using a simple novel tool. Asian Spine J. (2021) 15:415–23. 10.31616/asj.2020.0366 33355845 PMC8377218

[103] Miyagi M. ISSLS Prize winner: Increased innervation and sensory nervous system plasticity in a mouse model of low back pain due to intervertebral disc degeneration. Spine. (2014) 39:1345–54. 10.1097/BRS.0000000000000334 24718079

[104] Millecamps M Czerminski JT Mathieu AP Stone LS. Behavioral signs of axial low back pain and motor impairment correlate with the severity of intervertebral disc degeneration in a mouse model. Spine J. (2015) 15:2524–37. 10.1016/j.spinee.2015.08.055 26334234

[105] Millecamps M Tajerian M Sage EH Stone LS. Behavioral signs of chronic back pain in the SPARC-null mouse. Spine. (2011) 36:95–102. 10.1097/BRS.0b013e3181cd9d75 20714283 PMC3007098

[106] Kudelko M Chen P Tam V Zhang Y Kong O-Y Sharma R. PRIMUS: Comprehensive proteomics of mouse intervertebral discs that inform novel biology and relevance to human disease modelling. Matrix Biol Plus. (2021) 12:100082. 10.1016/j.mbplus.2021.100082 34409283 PMC8361275

[107] Richardson SM Ludwinski FE Gnanalingham KK Atkinson RA Freemont AJ Hoyland JA. Notochordal and nucleus pulposus marker expression is maintained by sub-populations of adult human nucleus pulposus cells through aging and degeneration. Sci Rep. (2017) 7:1–11. 10.1038/s41598-017-01567-w 28473691 PMC5431421

[108] Zhang Y Xiong C Kudelko M Li Y Wang C Wong YL. Early onset of disc degeneration in SM/J mice is associated with changes in ion transport systems and fibrotic events. Matrix Biol. (2018) 70:123–39. 10.1016/j.matbio.2018.03.024 29649547

[109] Urban JPGG Roberts S. Degeneration of the intervertebral disc. Arthritis Res Ther. (2003) 5:120–30. 10.1186/ar629 12723977 PMC165040

[110] Roberts S Evans H Trivedi J Menage J. Histology and pathology of the human intervertebral disc. J Bone Jt Surg. (2006) 88:10–4. 10.2106/00004623-200604002-00003 16595436

[111] Risbud M V Schoepflin ZR Mwale F Kandel RA Grad S Iatridis JC. Defining the phenotype of young healthy nucleus pulposus cells: Recommendations of the Spine Research Interest Group at the 2014 annual ORS meeting. J Orthop Res. (2015) 33:283–93. 10.1002/jor.22789 25411088 PMC4399824

[112] Cloyd JM Elliott DM. Elastin content correlates with human disc degeneration in the anulus fibrosus and nucleus pulposus. Spine. (2007) 32:1826–31. 10.1097/BRS.0b013e3181132a9d 17762289

[113] Yoshimoto Y Takimoto A Watanabe H Hiraki Y Kondoh G Shukunami C. Scleraxis is required for maturation of tissue domains for proper integration of the musculoskeletal system. Sci Rep. (2017) 7:45010. 10.1038/srep45010 28327634 PMC5361204

[114] Nakamichi R Ito Y Inui M Onizuka N Kayama T Kataoka K. Mohawk promotes the maintenance and regeneration of the outer annulus fibrosus of intervertebral discs. Nat Commun. (2016) 7:1–14. 10.1038/ncomms12503 27527664 PMC4990710

[115] Torre OM Mroz V Bartelstein MK Huang AH Iatridis JC. Annulus fibrosus cell phenotypes in homeostasis and injury: implications for regenerative strategies. Ann N Y Acad Sci. (2019) 1442:61–78. 10.1111/nyas.13964 30604562 PMC6417974

[116] Kadow T Sowa G Vo N Kang JD. Molecular Basis of Intervertebral Disc Degeneration and Herniations: What Are the Important Translational Questions? Clin Orthop Relat Res. (2015) 473:1903–12. 10.1007/s11999-014-3774-8 25024024 PMC4418989

[117] Adams MA Roughley PJ MA Adams PR Adams MA Roughley PJ. What is intervertebral disc degeneration, and what causes it? Spine. (2006) 31:2151–61. 10.1097/01.brs.0000231761.73859.2c 16915105

[118] Brendler J Winter K Lochhead P Schulz A Ricken AM. Histological differences between lumbar and tail intervertebral discs in mice. J Anat. (2022) 240:84–93. 10.1111/joa.13540 34427936 PMC8655214

[119] Zhang Y Tian Z Gerard D Yao L Shofer FS Cs-Szabo G. Elevated inflammatory gene expression in intervertebral disc tissues in mice with ADAM8 inactivated. Sci Rep. (2021) 11:1–11. 10.1038/s41598-021-81495-y 33469101 PMC7815795

[120] Yan Z Yin L Wang Z Ye J Zhang Z Li R. A novel organ culture model of mouse intervertebral disc tissues. Cells Tissues Organs. (2015) 201:38–50. 10.1159/000439268 26447649 PMC4710565

[121] Cao Y Liao S Zeng H Ni S Tintani F Hao Y. 3D characterization of morphological changes in the intervertebral disc and endplate during aging: a propagation phase contrast synchrotron micro-tomography study. Sci Rep. (2017) 7:1–12. 10.1038/srep43094 28266560 PMC5339826

[122] Leung VYL Chan WCW Hung SC Cheung KMC Chan D. Matrix remodeling during intervertebral disc growth and degeneration detected by multichromatic fast staining. J Histochem Cytochem. (2009) 57:249–56. 10.1369/jhc.2008.952184 19001641 PMC2664937

[123] Tam V Chan WCW Leung VYL Cheah KSE Cheung KMC Sakai D. Histological and reference system for the analysis of mouse intervertebral disc. J Orthop Res. (2018) 36:233–43. 10.1002/jor.23637 28636254

[124] Liu H Qian BP Qiu Y Wang Y Wang B Yu Y. Vertebral body or intervertebral disc wedging: Which contributes more to thoracolumbar kyphosis in ankylosing spondylitis patients? a retrospective study. Med. (2016) 95:e4855. 10.1097/MD.0000000000004855 27661026 PMC5044896

[125] Pfirrmann CCWA Metzdorf A Zanetti M Hodler J Boos N. Magnetic resonance classification of lumbar intervertebral disc degeneration. Spine. (2001) 26:1873–8. 10.1097/00007632-200109010-00011 11568697

[126] Torre OM Das R Berenblum RE Huang AH Iatridis JC. Neonatal mouse intervertebral discs heal with restored function following herniation injury. FASEB J. (2018) 32:4753–62. 10.1096/fj.201701492R 29570392 PMC6103171

[127] Liu JW Abraham AC Tang SY. The high-throughput phenotyping of the viscoelastic behavior of whole mouse intervertebral discs using a novel method of dynamic mechanical testing. J Biomech. (2015) 48:2189–94. 10.1016/j.jbiomech.2015.04.040 26004435 PMC4492880

[128] Nakahara H Hasegawa A Otabe K Ayabe F Matsukawa T Onizuka N. Transcription factor Mohawk and the pathogenesis of human anterior cruciate ligament degradation. Arthritis Rheum. (2013) 65:2081–9. 10.1002/art.38020 23686683 PMC3840305

[129] Melgoza IP Chenna SS Tessier S Zhang Y Tang SY Ohnishi T. Development of a standardized histopathology scoring system using machine learning algorithms for intervertebral disc degeneration in the mouse model-An ORS spine section initiative. JOR spine. (2021) 4:e1164. 10.1002/jsp2.1164 34337338 PMC8313179

[130] Le Maitre CL Dahia CL Giers M Illien-Junger S Cicione C Samartzis D. Development of a standardized histopathology scoring system for human intervertebral disc degeneration: an Orthopaedic Research Society Spine Section Initiative. JOR Spine. (2021) 4:e1167. 10.1002/jsp2.1167 34337340 PMC8313169

[131] Palmer EI Lotz JC. The compressive creep properties of normal and degenerated murine intervertebral discs. J Orthop Res. (2004) 22:164–9. 10.1016/S0736-0266(03)00161-X 14656676

[132] Shapiro IM Risbud MV. The Intervertebral Disc. Vienna: Springer Vienna (2014).

[133] Lai A Moon A Purmessur D Skovrlj B Winkelstein BA Cho SK. Assessment of functional and behavioral changes sensitive to painful disc degeneration. J Orthop Res. (2015) 33:755–64. 10.1002/jor.22833 25731955 PMC4406864

[134] Cornejo MCC Cho SKK Giannarelli C Iatridis JCC Purmessur D. Soluble factors from the notochordal-rich intervertebral disc inhibit endothelial cell invasion and vessel formation in the presence and absence of pro-inflammatory cytokines. Osteoarthr Cartil. (2015) 23:487–96. 10.1016/j.joca.2014.12.010 25534363 PMC4411226

[135] Richardson SM Purmessur D Baird P Probyn B Freemont AJ Hoyland JA. Degenerate human nucleus pulposus cells promote neurite outgrowth in neural cells. PLoS ONE. (2012) 7:e47735. 10.1371/journal.pone.0047735 23091643 PMC3472988

[136] Freemont AJ Peacock TE Goupille P Hoyland JA O'Brien J Jayson MIV. Nerve ingrowth into diseased intervertebral disc in chronic back pain. Lancet. (1997) 350:178–81. 10.1016/S0140-6736(97)02135-1 9250186

[137] Thompson JP Pearce RH Schechter MT Adams ME Tsang IKY Bishop PB. Preliminary evaluation of a scheme for grading the gross morphology of the human intervertebral disc. Spine. (1990) 15:411–5. 10.1097/00007632-199005000-00012 2363069

[138] Sone Y Kakuta M Misaki A. Isolation and characterization of polysaccharides of “kikurage,” fruit body of auricularia auricula-judae. Agric Biol Chem. (1978) 42:417–25. 10.1080/00021369.1978.10862990

[139] Showalter BL Beckstein JC Martin JT Beattie EE Orías AAE Schaer TP. Comparison of animal discs used in disc research to human lumbar disc. Spine. (2012) 37:E900–7. 10.1097/BRS.0b013e31824d911c 22333953 PMC3377819

[140] Beckstein JC Sen S Schaer TP Vresilovic EJ Elliott DM. Comparison of animal discs used in disc research to human lumbar disc. Spine. (2008) 33:E166–73. 10.1097/BRS.0b013e318166e001 18344845

[141] Sarver JJ Elliott DM. Altered disc mechanics in mice genetically engineered for reduced type I collagen. Spine. (2004) 29:1094–8. 10.1097/00007632-200405150-00009 15131436

[142] Elliott DM Sarver JJ. Young investigator award winner: validation of the mouse and rat disc as mechanical models of the human lumbar disc. Spine. (2004) 29:713–22. 10.1097/01.BRS.0000116982.19331.EA 15087791

[143] Chiu EJ. Characterization of the human intervertebral disc with magnetic resonance imaging. University of California, San Francisco (1998). Available online at: https://www.proquest.com/openview/b2efafca3808123f605bf4acf76160b9/1?pq-origsite=gscholar&cbl=18750&diss=y

[144] Brinjikji W Diehn FE Jarvik JG Carr CM Kallmes DF Murad MH. Findings of disc degeneration are more prevalent in adults with low back pain than in asymptomatic controls: a systematic review and meta-analysis. Am J Neuroradiol. (2015) 36:2394–9. 10.3174/ajnr.A4498 26359154 PMC7964277

[145] Saab CY. Chronic Pain and Brain Abnormalities. London: Academic Press (2014). p. 1–148.

[146] Dubin AE Patapoutian A. Nociceptors: the sensors of the pain pathway. J Clin Invest. (2010) 120:3760–72. 10.1172/JCI42843 21041958 PMC2964977

[147] Zhang S Hu B Liu W Wang P Lv X Chen S. The role of structure and function changes of sensory nervous system in intervertebral disc-related low back pain. Osteoarthr Cartil. (2021) 29:17–27. 10.1016/j.joca.2020.09.002 33007412

[148] Fitzcharles MA Cohen SP Clauw DJ Littlejohn G Usui C Häuser W. Nociplastic pain: towards an understanding of prevalent pain conditions. Lancet. (2021) 397:2098–110. 10.1016/S0140-6736(21)00392-5 34062144

[149] Pengel LHM Herbert RD Maher CG Refshauge KM. Acute low back pain: systematic review of its prognosis. BMJ. (2003) 327:323. 10.1136/bmj.327.7410.323 12907487 PMC169642

[150] Low Back Pain Fact Sheet | National Institute of Neurological Disorders and Stroke. Available online at: https://www.ninds.nih.gov/Disorders/Patient-Caregiver-Education/Fact-Sheets/Low-Back-Pain-Fact-Sheet (accessed January 13, 2022).

[151] Van Tulder M Becker A Bekkering T Breen A Del Real MTG Hutchinson A. Chapter 3 European guidelines for the management of acute nonspecific low back pain in primary care. Eur Spine J. (2006) 15:s169–91. 10.1007/s00586-006-1071-2 16550447 PMC3454540

[152] Deuis JR Dvorakova LS Vetter I. Methods used to evaluate pain behaviors in rodents. Front Mol Neurosci. (2017) 10:284. 10.3389/fnmol.2017.00284 28932184 PMC5592204

[153] Bogduk N. On the definitions and physiology of back pain, referred pain, and radicular pain. Pain. (2009) 147:17–9. 10.1016/j.pain.2009.08.020 19762151

[154] Devereaux M. Low back pain. Med Clin North Am. (2009) 93:477–501. 10.1016/j.mcna.2008.09.013 19272519

[155] Ray P Torck A Quigley L Wangzhou A Neiman M Rao C. Comparative transcriptome profiling of the human and mouse dorsal root ganglia: an RNA-seq–based resource for pain and sensory neuroscience research. Pain. (2018) 159:1325–45. 10.1097/j.pain.0000000000001217 29561359 PMC6008200

[156] García-Cosamalón J del Valle ME Calavia MG García-Suárez O López-Muñiz A Otero J. Intervertebral disc, sensory nerves and neurotrophins: who is who in discogenic pain? J Anat. (2010) 217:1–15. 10.1111/j.1469-7580.2010.01227.x 20456524 PMC2913007

[157] Purmessur D Cornejo MC Cho SK Roughley PJ Linhardt RJ Hecht AC. Intact glycosaminoglycans from intervertebral disc-derived notochordal cell-conditioned media inhibit neurite growth while maintaining neuronal cell viability. Spine J. (2015) 15:1060–9. 10.1016/j.spinee.2015.02.003 25661435 PMC4416992

[158] Stefanakis M Al-Abbasi M Harding I Pollintine P Dolan P Tarlton J. Annulus fissures are mechanically and chemically conducive to the ingrowth of nerves and blood vessels. Spine. (2012) 37:1883–91. 10.1097/BRS.0b013e318263ba59 22706090

[159] LA Binch A Cole AA Breakwell LM Michael ALR Chiverton N Cross AK. Expression and regulation of neurotrophic and angiogenic factors during human intervertebral disc degeneration. Arthritis Res Ther. (2014) 16:416. 10.1186/s13075-014-0416-1 25209447 PMC4177417

[160] Binch ALA Cole AA Breakwell LM Michael ALR Chiverton N Creemers LB. Nerves are more abundant than blood vessels in the degenerate human intervertebral disc. Arthritis Res Ther. (2015) 17:370. 10.1186/s13075-015-0889-6 26695177 PMC4704545

[161] Bennett DL Clark XAJ Huang J Waxman SG Dib-Hajj SD. The Role of Voltage-Gated Sodium Channels in Pain Signaling. Physiol Rev. (2019) 99:1079–151. 10.1152/physrev.00052.2017 30672368

[162] Leimer EM Gayoso MG Jing L Tang SY Gupta MC Setton LA. Behavioral compensations and neuronal remodeling in a rodent model of chronic intervertebral disc degeneration. Sci Rep. (2019) 9:1–10. 10.1038/s41598-019-39657-6 30842475 PMC6403208

[163] Dutta S Sengupta P. Men and mice: relating their ages. Life Sci. (2016) 152:244–8. 10.1016/j.lfs.2015.10.025 26596563

[164] Aoki Y Ohtori S Takahashi K Ino H Douya H Ozawa T. Expression and co-expression of VR1, CGRP, and IB4-binding glycoprotein in dorsal root ganglion neurons in rats: differences between the disc afferents and the cutaneous afferents. Spine. (2005) 30:1496–500. 10.1097/01.brs.0000167532.96540.31 15990662

[165] Ohtori S Takahashi K Moriya H. Existence of brain-derived neurotrophic factor and vanilloid receptor subtype 1 immunoreactive sensory DRG neurons innervating L5/6 intervertebral discs in rats. J Orthop Sci. (2003) 8:84–7. 10.1007/s007760300014 12560892

[166] Rush AM Cummins TR Waxman SG. Multiple sodium channels and their roles in electrogenesis within dorsal root ganglion neurons. J Physiol. (2007) 579:1–14. 10.1113/jphysiol.2006.121483 17158175 PMC2075388

[167] Teichert RW Smith NJ Raghuraman S Yoshikami D Light AR Olivera BM. Functional profiling of neurons through cellular neuropharmacology. Proc Natl Acad Sci U S A. (2012) 109:1388–95. 10.1073/pnas.1118833109 22307590 PMC3277115

[168] Hassler SN Kume M Mwirigi JM Ahmad A Shiers S Wangzhou A. The cellular basis of protease-activated receptor 2–evoked mechanical and affective pain. JCI Insight. (2020) 5:e137393. 10.1172/jci.insight.137393 32352932 PMC7308051

[169] Miyagi M. ISSLS prize winner: disc dynamic compression in rats produces long-lasting increases in inflammatory mediators in discs and induces long-lasting nerve injury and regeneration of the afferent fibers innervating discsa pathomechanism for chronic discogenic low back pain. Spine. (2012) 37:1810–8. 10.1097/BRS.0b013e31824ffac6 22366969

[170] Tappe-Theodor A Kuner R. Studying ongoing and spontaneous pain in rodents–challenges and opportunities. Eur J Neurosci. (2014) 39:1881–90. 10.1111/ejn.12643 24888508

[171] Mogil JS Crager SE. What should we be measuring in behavioral studies of chronic pain in animals? Pain. (2004) 112:12–5. 10.1016/j.pain.2004.09.028 15494180

[172] Gracely RH Grant MAB Giesecke T. Evoked pain measures in fibromyalgia. Best Pract Res Clin Rheumatol. (2003) 17:593–609. 10.1016/S1521-6942(03)00036-6 12849714

[173] Terminology | International Association for the Study of Pain. Available online at: https://www.iasp-pain.org/resources/terminology/?ItemNumber=1698&navItemNumber=576#pain (accessed January 13, 2022).

[174] Denenberg VH. Open-field behavior in the rat. What does it mean? Ann N Y Acad Sci. (1969) 159:852–9. 10.1111/j.1749-6632.1969.tb12983.x 5260302

[175] Langford DJ Bailey AL Chanda ML Clarke SE Drummond TE Echols S. Coding of facial expressions of pain in the laboratory mouse. Nat Methods. (2010) 7:447–9. 10.1038/nmeth.1455 20453868

[176] Deacon RMJ. Burrowing in rodents: a sensitive method for detecting behavioral dysfunction. Nat Protoc. (2006) 1:118–21. 10.1038/nprot.2006.19 17406222

[177] D'Amour FE Smith DL. A method for determining loss of pain sensation. J Pharmacol Exp Ther. (1941) 72:74–9.

[178] Banik RK Kabadi RA. A modified Hargreaves' method for assessing threshold temperatures for heat nociception. J Neurosci Methods. (2013) 219:41–51. 10.1016/j.jneumeth.2013.06.005 23796910 PMC3759573

[179] Deuis JR Yin K Cooper MA Schroder K Vetter I. Role of the NLRP3 inflammasome in a model of acute burn-induced pain. Burns. (2017) 43:304–9. 10.1016/j.burns.2016.09.001 28040362

[180] Deuis JR Vetter I. The thermal probe test: A novel behavioral assay to quantify thermal paw withdrawal thresholds in mice. Temperature. (2016) 3:199–207. 10.1080/23328940.2016.1157668 27857950 PMC4965000

[181] Yoon C Young Wook Y Heung Sik N Sun Ho K Jin Mo C. Behavioral signs of ongoing pain and cold allodynia in a rat model of neuropathic pain. Pain. (1994) 59:369–76. 10.1016/0304-3959(94)90023-X 7708411

[182] Brenner DS Golden JP Gereau RW. A Novel Behavioral Assay for Measuring Cold Sensation in Mice. PLoS ONE. (2012) 7:e39765. 10.1371/journal.pone.0039765 22745825 PMC3382130

[183] Kehl LJ Trempe TM Hargreaves KM. A new animal model for assessing mechanisms and management of muscle hyperalgesia. Pain. (2000) 85:333–43. 10.1016/S0304-3959(99)00282-1 10781907

[184] Putten MV Aartsma-Rus A Louvain L. The use of hanging wire tests to monitor muscle strength and condition over time. Treat-NmdEu. (2012) 1–12.

[185] Steru L Chermat R Thierry B Simon P. The tail suspension test: a new method for screening antidepressants in mice. Psychopharmacology. (1985) 85:367–70. 10.1007/BF00428203 3923523

[186] Jones BJ Roberts DJ. The quantitative measurement of motor inco-ordination in naive mice using an accelerating rotarod. J Pharm Pharmacol. (2011) 20:302–4. 10.1111/j.2042-7158.1968.tb09743.x 4384609

[187] Pitts M. Barnes maze procedure for spatial learning and memory in mice. Bio-protocol. (2018) 8:e2744. 10.21769/BioProtoc.2744 29651452 PMC5891830

[188] Schiltenwolf M Akbar M Neubauer E Gantz S Flor H Hug A. The cognitive impact of chronic low back pain: Positive effect of multidisciplinary pain therapy. Scand J pain. (2017) 17:273–8. 10.1016/j.sjpain.2017.07.019 28993113

[189] Krock E Millecamps M Currie JB Stone LS Haglund L. Low back pain and disc degeneration are decreased following chronic toll-like receptor 4 inhibition in a mouse model. Osteoarthr Cart. (2018) 26:1236–46. 10.1016/j.joca.2018.06.002 29908959

[190] Mogil JS. The translatability of pain across species. Philos Trans R Soc Lond B Biol Sci. (2019) 374:20190286. 10.1098/rstb.2019.0286 31544615 PMC6790385

[191] Mogil JS. Animal models of pain: progress and challenges. Nat Rev Neurosci. (2009) 10:283–94. 10.1038/nrn2606 19259101

[192] Averill S McMahon SB Clary DO Reichardt LF Priestley JV. Immunocytochemical localization of trka receptors in chemically identified subgroups of adult rat sensory neurons. Eur J Neurosci. (1995) 7:1484–94. 10.1111/j.1460-9568.1995.tb01143.x 7551174 PMC2758238

[193] Palmgren T Grönblad M Virri J Kääpä E Karaharju E. An immunohistochemical study of nerve structures in the anulus fibrosus of human normal lumbar intervertebral discs. Spine. (1999) 24:2075–9. 10.1097/00007632-199910150-00002 10543001

[194] Ahmed M Bjurholm A Kreicbergs A Schultzberg M. Neuropeptide Y, tyrosine hydroxylase and vasoactive intestinal polypeptide-immunoreactive nerve fibers in the vertebral bodies, discs, dura mater, and spinal ligaments of the rat lumbar spine. Spine. (1993) 18:268–73. 10.1097/00007632-199302000-00016 8095104

[195] Wu DF Chandra D McMahon T Wang D Dadgar J Kharazia VN. PKCε phosphorylation of the sodium channel NaV18 increases channel function and produces mechanical hyperalgesia in mice. J Clin Invest. (2012) 122:1306–15. 10.1172/JCI61934 22426212 PMC3315445

[196] Laedermann CJ Abriel H Decosterd I. Post-translational modifications of voltage-gated sodium channels in chronic pain syndromes. Front Pharmacol. (2015) 6:263. 10.3389/fphar.2015.00263 26594175 PMC4633509

[197] Rostock C Schrenk-Siemens K Pohle J Siemens J. Human vs. Mouse nociceptors – similarities and differences. Neuroscience. (2018) 387:13–27. 10.1016/j.neuroscience.2017.11.047 29229553 PMC6150929

[198] Middleton SJ Barry AM Comini M Li Y Ray PR Shiers S. Studying human nociceptors: from fundamentals to clinic. Brain. (2021) 144:1312–35. 10.1093/brain/awab048 34128530 PMC8219361

[199] Han C Estacion M Huang J Vasylyev D Zhao P Dib-Hajj SD. Human Nav18: Enhanced persistent and ramp currents contribute to distinct firing properties of human DRG neurons. J Neurophysiol. (2015) 113:3172–85. 10.1152/jn.00113.2015 25787950 PMC4432682

[200] Lee JH An JH Lee SH Seo IS. Three-dimensional gait analysis of patients with weakness of ankle dorsiflexor as a result of unilateral L5 radiculopathy. J Back Musculoskelet Rehabil. (2010) 23:49–54. 10.3233/BMR-2010-0248 20555116

[201] O'Sullivan P. Diagnosis and classification of chronic low back pain disorders: maladaptive movement and motor control impairments as underlying mechanism. Man Ther. (2005) 10:242–55. 10.1016/j.math.2005.07.001 16154380

[202] LaCroix-Fralish ML Rutkowski MD Weinstein JN Mogil JS DeLeo JA. The magnitude of mechanical allodynia in a rodent model of lumbar radiculopathy is dependent on strain and sex. Spine. (2005) 30:1821–7. 10.1097/01.brs.0000174122.63291.38 16103850

[203] Nygaard OP Mellgren SI. The function of sensory nerve fibers in lumbar radiculopathy. Use of quantitative sensory testing in the exploration of different populations of nerve fibers and dermatomes. Spine. (1998) 23:348–52. 10.1097/00007632-199802010-00012 9507624

[204] Borenstein DG. Epidemiology, etiology, diagnostic evaluation, and treatment of low back pain. Curr Opin Rheumatol. (2000) 12:143–9. 10.1097/00002281-200003000-00008 10751017

[205] Winter CC Brandes M Müller C Schubert T Ringling M Hillmann A. Walking ability during daily life in patients with osteoarthritis of the knee or the hip and lumbar spinal stenosis: a cross sectional study. BMC Musculoskelet Disord. (2010) 11:233. 10.1186/1471-2474-11-233 20939866 PMC2958990

[206] Mitchell K Porter M Anderson L Phillips C Arceo G Montz B. Differences in lumbar spine and lower extremity kinematics in people with and without low back pain during a step-up task: a cross-sectional study. BMC Musculoskelet Disord. (2017) 18:369. 10.1186/s12891-017-1721-z 28841866 PMC5574078

[207] Renthal W Chamessian A Curatolo M Davidson S Burton M Dib-Hajj S. Human cells and networks of pain: transforming pain target identification and therapeutic development. Neuron. (2021) 109:1426–9. 10.1016/j.neuron.2021.04.005 33957072 PMC9208579

[208] Krock E Millecamps M Anderson KM Srivastava A Reihsen TE Hari P. Interleukin-8 as a therapeutic target for chronic low back pain: upregulation in human cerebrospinal fluid and pre-clinical validation with chronic reparixin in the SPARC-null mouse model. EBioMedicine. (2019) 43:487–500. 10.1016/j.ebiom.2019.04.032 31047862 PMC6558025

[209] Kim Y. Prediction of peripheral tears in the anulus of the intervertebral disc. Spine. (2000) 25:1771–4. 10.1097/00007632-200007150-00006 10888944

[210] Wang YXJ Griffith JF Zeng XJ Deng M Kwok AWL Leung JCS. Prevalence and sex difference of lumbar disc space narrowing in elderly chinese men and women: osteoporotic fractures in men (Hong Kong) and osteoporotic fractures in women (Hong Kong) studies. Arthritis Rheum. (2013) 65:1004–10. 10.1002/art.37857 23335175 PMC3618501

[211] Mosley GE Hoy RC Nasser P Kaseta T Lai A Evashwick-Rogler TW. Sex differences in rat intervertebral disc structure and function following annular puncture injury. Spine. (2019) 44:1257–69. 10.1097/BRS.0000000000003055 30973506 PMC6722021

[212] Wáng YXJ Wáng JQ Káplár Z. Increased low back pain prevalence in females than in males after menopause age: evidences based on synthetic literature review. Quant Imaging Med Surg. (2016) 6:199–206. 10.21037/qims.2016.04.06 27190772 PMC4858456

